# Dual roles of syndecan-4 in regulating chicken fibrosis *in vitro*

**DOI:** 10.3389/fphys.2026.1782914

**Published:** 2026-03-23

**Authors:** Lucie Pejšková, Nina Therese Solberg, Marianne Lunde, Cathrine Rein Carlson, Mona Elisabeth Pedersen, Sissel Beate Rønning

**Affiliations:** 1 Nofima AS, Raw Materials and Optimization, Ås, Norway; 2 Institute for Experimental Medical Research, Oslo University Hospital and University of Oslo, Oslo, Norway

**Keywords:** broiler chicken, fibroblasts, fibrosis, syndecan-4, wooden breast

## Abstract

**Introduction:**

Wooden Breast (WB) is a myopathy affecting the skeletal breast muscle (*Pectoralis major*) in broiler chickens and is characterized by muscle fiber damage and varying degrees of fibrosis, ECM remodeling and inflammation. Several key factors such as pro-inflammatory cytokines like TGF-β1 and IL-1β, drive fibrosis in WB myopathy. We have previously shown that the expression of syndecan-4 (SDC4), a transmembrane proteoglycan, was increased in WB poultry skeletal muscle tissue. Furthermore, the ectodomain shedding of SDC4 by matrix metalloproteinases (MMPs) differed in the skeletal muscle satellite cells from isolated affected chickens compared with normal. While SDC4 has been previously implicated as a key driver for regulating myofibroblast activity in mechanically induced fibrosis in cardiac tissue, its specific role and shedding activity in chicken fibroblasts in relation to WB myopathy remain poorly understood.

**Methods:**

*In vitro* the overexpression system was used to mimic the previously detected increased SDC4 levels in WB and to further investigate fibrotic markers and syndecans at the gene and protein levels. Furthermore, we used blocking peptides derived from the SDC4 ectodomain and investigated their effect on SDC4 shedding and fibrotic markers. Additionally, TGF-β1 treatment, the main trigger of myofibroblasts, fibrosis, and cytokines, was used to investigate the connection between SDC4 shedding and fibrosis.

**Results and discussion:**

Overexpression of *SDC4* in chicken fibroblasts reduced the gene and protein expression of fibrotic markers such as collagen I, collagen III and MMP-2. At the same time, we observed an increase in the gene expression of *TGFB1* and *IL1B*. *SDC4* overexpression also modulated intracellular proteins connected to fibrosis-relevant signaling pathways, with increased phosphorylation of p38 MAPK and decreased phosphorylation of Akt. Moreover, we could observe a decreased production of ribosomal protein S6 and β-catenin. *SDC4* overexpression induced shedding of a 15 kDa SDC4 fragment, while a 20 kDa fragment was produced at similar level regardless of overexpression. Modulation of ectodomain shedding using blocking peptides targeting various ectodomain regions (amino acids 76–106 and 100–130) significantly reduced the production of the 20 kDa endogenous fragment. However, it did not affect the expression level of the 15 kDa fragment. This contrasts with what we have seen with the same blocking peptides in chicken skeletal muscle satellite cells where the expression level of the 15 kDa fragment was reduced. When we stimulated the cells with TGF-β1, an increase in gene expression of fibrotic marker was observed as expected. No change in SDC4 gene expression was observed, while SDC4 fragment of 20 kDa was increased. Taken together, our results reveal a complex role of SDC4 and shedding in modulating fibrotic responses in chicken fibroblasts, suggesting a potential dual function where the full-length SDC4, produced by overexpression, may act as an anti-fibrotic regulator, while the TGF-β1 induced shed ectodomain fragments could promote pro-fibrotic and inflammatory processes.

## 1 Introduction

The efforts to enhance chicken meat production by increasing the growth rate have led to the development of production-associated diseases. Among these are pectoral muscle myopathies like wooden breast (WB) (Petracci et al., 2015). WB-affected muscles are characterized by visible hardening, pale discoloration, and increased rigidity (Sihvo et al., 2014). Microscopically, WB is defined by massive infiltration of extracellular matrix (fibrosis), extensive myofiber degeneration, necrosis, infiltration of inflammatory cells, and lipidosis (Papah et al., 2017) compromising the functional integrity of the muscle and reducing meat quality (Wold et al., 2019; Geronimo et al., 2021).

Fibrosis in general is driven by the activation of fibroblasts into myofibroblasts, leading to the production and remodeling of ECM proteins, such as collagen and decorin (Frangogiannis, 2016; Velleman et al., 2017). This excessive ECM accumulation stiffens the tissue, replacing functional muscle fibers with non-contractile connective tissue. The overproduction and crosslinking of ECM components, especially collagens, disrupts muscle architecture and exacerbates mechanical stress on surrounding tissues, further perpetuating the disease process (Sihvo et al., 2014; Velleman, 2023). Fibrosis arises from a complex interplay of cellular responses and signaling pathways such as TGF-β, Wnt/β-catenin, MAPK, and PI3K/Akt, which collectively drive fibroblast activation, ECM deposition, and tissue remodeling. TGF-β acts as a master regulator by activating SMAD-dependent and non-SMAD signaling cascades, including MAPK and PI3K/Akt. At the same time, the Wnt/β-catenin pathway stabilizes β-catenin, promoting the transcription of fibrotic genes (Biernacka et al., 2011). MAPK pathways, such as ERK and p38, amplify pro-fibrotic signals and enhance fibroblast proliferation and survival (Foglia et al., 2019).

Syndecans are transmembrane proteoglycans that regulate cell adhesion, migration, and signaling by interacting with the ECM and growth factors (Couchman, 2010). The family includes four members: syndecan-1, -2, -3, and -4, each with distinct roles in tissue repair and remodeling (Chung et al., 2016). A conserved transmembrane domain, a short cytoplasmic domain with a unique variable region specific to each syndecan, and a large, diverse extracellular domain with glycosaminoglycans (GAG) chains characterize each syndecan. Among them, syndecan-4 (SDC4) plays important role in fibrosis and connects different signaling pathways (Jiang et al., 2010; Elfenbein and Simons, 2013; Tanino et al., 2019; Herum et al., 2020; Gopal et al., 2021; Onyeisi et al., 2021; Pejšková et al., 2023; Strand et al., 2023). SDC4 is the most ubiquitously expressed member of the SDC family and plays a general role in adhesion, migration, and responses to mechanical stress. SDC4 appears protective in fibrotic disease models, as its absence is often associated with deteriorated outcomes (Gopal et al., 2021). SDC4 shedding can be mediated by enzymes such as matrix metalloproteinases (MMPs) −2 and −9 (Manon-Jensen et al., 2013). This process releases the ectodomain of SDC4 into the ECM, where it can bind to cytokines and influence cell signaling. Shedding can have multiple effects on cell signaling: it can downregulate surface signal transduction, quench ligands and make them unavailable to the remaining SDCs, or initiate additional signaling pathways as circulating soluble effectors, independent of cell surface signaling (Gopal, 2020). The shedding of SDC4 happens in basal metabolism but is increased during different pathologies (Bertrand and Bollmann, 2019), including fibrosis (Herum et al., 2020) and WB myopathy (Pejšková et al., 2023). Our previous research demonstrated that shedding of SDC4s ectodomain was altered in the skeletal muscle satellite cells from WB-affected chickens compared with normal (Pejšková et al., 2024). Shed SDC ectodomains are detectable in inflammatory fluids (Subramanian et al., 1997), which might mediate inflammation (Fitzgerald et al., 2000). Shed soluble ectodomains have been proposed as biomarkers, with significant research focusing on SDC1 (Bertrand and Bollmann, 2019). Elevated levels of shed SDC4 have previously been observed in heart failure (Bielecka-Dabrowa et al., 2015; Strand et al., 2015). However, the role of SDC4 shedding in the context of fibrosis in WB still needs to be better understood. Blocking the shedding of SDCs has emerged as a potential strategy for mitigating fibrosis (Bertrand and Bollmann, 2019). While studies on SDC1 have demonstrated the therapeutic potential of inhibiting its shedding (Tromp et al., 2014), the effects of blocking SDC4 shedding have been less explored. We have recently shown that peptides representing the SDC4 ectodomain can effectively reduce SDC4 shedding and attenuate the proliferation of *SDC4*-overexpressing chicken muscle satellite cells (Pejšková et al., 2024).

Several studies have highlighted the role of pro-inflammatory cytokines, including TGF-β and IL-1β in fibrosis through their interaction with SDC4 (Marshall et al., 2003; Strand et al., 2015; Tanino et al., 2019). Specifically, TGF-β is known for its interactions with heparan sulfate proteoglycans (Lu et al., 2014), including SDCs (Chen et al., 2004; Tanino et al., 2019). For instance, SDC4 expression is upregulated in fibrotic tissues such as cardiac and lung fibroblasts in response to TGF-β1 (Strand et al., 2013; Toba-Ichihashi et al., 2016). However, there is limited research of TGF-β1 on the role of SDC4 in chicken fibrosis and its impact on SDC4 shedding.

This study explores the role of SDC4 in chicken fibroblasts by examining: the effects of *SDC4* overexpression on (1) fibrotic processes, and (2) SDC4-associated signaling mechanisms, and the impact of (3) blocking peptides on SDC4 shedding and fibrosis, and (4) TGF-β1 on *SDC4* expression and fibrotic signaling. The findings reveal a complex role for SDC4, with the full-length protein potentially acting as an anti-fibrotic regulator, while its shed ectodomain fragments may promote pro-fibrotic and inflammatory responses. Finally, the pro-fibrotic TGF-β1 increased SDC4 shedding, although no effect was observed on the *SDC4* gene expression level.

## 2 Materials and methods

### 2.1 Cell culture and treatment

#### 2.1.1 Cell maintenance

The embryonic chicken fibroblast cell line SL-29 was purchased from ATCC (#CRL 1590, Manassas, VA). The cells were stored at −150 °C and later expanded into multiple aliquots, which were frozen at 3rd and 4th passage. Cells were maintained in growth medium (DMEM GlutaMAX, 4.5 g/L D-glucose [#61965–026, Gibco, Thermo fisher Scientific, Waltham, MA, United States], TPB [#18050–039, Gibco [5%]], FBS [10%, #F7524, Sigma-Aldrich, Darmstadt, Germany], and Penicilin/Streptomicin [1%]) in a humified atmosphere of 5% CO_2_ at 37 °C. All experiments were performed with cells at passage 6th or 7th, and cell counting was performed by NucleoCounter NC-202 (Chemometec A/S, Denmark).

#### 2.1.2 Transient transfection

Chicken embryonic fibroblasts SL-29 were seeded in growth medium without antibiotics at 300,000 cells per well in a six-well plate and left proliferating for 24 h to reach about 70% confluence. Before transfection, the growth medium was changed once. HA-syndecan-4 (HA-SDC4) and syndecan-4 (SDC4) (both separately cloned into plasmid pCEP4, custom-made by GenScript (Piscataway, NJ, United States), or the empty control vectors pEGFP-N1 (Genescript) and pCEP4 (#P49416, Thermo fisher Scientific, Waltham, MA, United States) were transfected into SL-29 using the Lipofectamine 3000 Transfection Kit (#L3000-008, Thermo Fisher Scientific). All transiently transfected cells were analyzed 24 h after transfection. All plasmids were separately transfected into cells with 2.5 μg of DNA per well. The transfection efficiency was measured by fluorescence 24 h after transfection of pEGFP-N1 in in the green channel of an Incucyte S3Live-Cell Analysis System (Sartorius AS, Germany), showing 32%–37% transfection efficiency (data not shown).

#### 2.1.3 Cytokines and blocking peptides treatments

Chicken embryonic fibroblasts SL-29 were seeded at 300,000 or 400,000 cells per well in a 6-well plate and left proliferating for 24 h at 37 °C, 5% CO_2_. Following this, the cells were either transiently transfected with SDC4 or treated with cytokines and then harvested after 24 h. Prior to cytokine treatments, cells were starved by growth medium lacking serum for 2 h. Cells were treated with cytokines (TGF-β1 #GF346, TNF-α #T6674, IL-6 #SRP3096, IL-1β #GF331, Sigma Merck, Darmstadt, Germany) at a concentration of 10 ng/mL, and with 100 μM LPS (#L4391, Sigma Merck) for 24 h before analysis. The concentration of cytokines was based on SDC4 research by Strand et al. (2013). Custom-made blocking peptides (BP) were developed based on the sequence of chicken SDC4 ectodomain (custom-made by GenScript). The BP and their corresponding solvents are described in (Pejšková et al., 2024). 24h after SDC4 transfection, an additional 2h of incubation with blocking peptides was performed. For all experiments, BPs were used at a concentration of 2.5 µM. Prior to RNA and protein harvesting, cells were rinsed twice with PBS before cell lysis with 350 µL of RLT buffer containing 0.04M DTT.

### 2.2 Western blotting

Sample preparation and procedure for detection of SDC4 protein were performed as previously described in (Pejšková et al., 2024). The protein expression detected by western blotting for other proteins in this study was conducted with the following modifications:

Cells were lysed on ice for 30 min in RIPA buffer (#89900, Thermo Fisher Scientific, Waltham, MA, United States) supplemented with phosphatase inhibitors (#04906845001, Sigma Merck, Darmstadt, Germany) and protease inhibitors (#04693132001, Roche). Lysates were subsequently centrifuged at 13,300 RCF speed for 15 min at 4 °C. The supernatant was collected, and protein concentrations were determined using the Bradford assay (5000006, Bio-Rad, Hercules. CA, United States). Each sample was prepared by mixing 15 µg of protein with 5 µL 4× LDS Sample Buffer (#NP0007, NuPAGE, Invitrogen) and 1 µL of Sample Reducing Agent (#NP0009, NuPAGE, Invitrogen) to a final volume of 20 µL. Samples were denatured at 70 °C for 10 min. Proteins were separated using NuPAGE 4%–12% Bis-Tris gels (#NP0322BOX, Invitrogen) in MOPS SDS Running Buffer (#NP0001, Invitrogen) at 200 V for approximately 50 min. After separation, the proteins were transferred to nitrocellulose membranes (either iBlot3 Transfer Stacks Midi NC [#IB33001, Invitrogen], or iBlot Gel Transfer Stacks, Regular [#IB301001, Invitrogen]) using the iBlot3 transfer system at 25 V for 6–8 min under low cooling conditions. Membranes were blocked in 2% ECL Prime Blocking Agent (#RPN418V, Cytiva, Marlborough, MA, United States) dissolved in TBS-T (0.1% Tween-20) for 1 h at room temperature. Primary antibodies were diluted in 0.2% ECL blocking solution in TBS-T and incubated with the membranes overnight at 4 °C. The following primary antibodies were used: Syndecan-4 (#429716, rabbit, 1:1000, custom made by GenScript, Piscataway, NJ, United States) as previously described in Pejšková*, et al., 2023*, including specificity of the detected bands. GAPDH (#sc-47724, mouse, 1:500, Santa Cruz Biotechnology, Dallas, TX, United States), MMP-2 (#ab181286, rabbit, 1:1000, abcam, Cambridge, UK), MMP-9 (#NBP1-57940, rabbit, 1:500, Bio-techne, Minneapolis, MI, United States), SMA (#ab5694, rabbit, 1:500, abcam), HA antibody (#32–6700, mouse, 1:250, Thermo Fisher Scientific), β-Catenin (#610153, mouse, 1:2000, BD Transduction Laboratories, Franklin Lakes, NJ, United States), non-phospho (Active) β-Catenin (ser33/37/Thr41) (D13A1) (#8814, rabbit, Cell Signaling), p44/42 MAPK (Erk1/2) (rabbit, #4695, 1:1000, Cell signaling), Phospho-p44/42 MAPK (Erk1/2) (Thr202/Tyr204) (rabbit, Cell Signaling, x9101, 1:1000), Akt (#9272, rabbit, 1:1000, Cell Signaling, Danvers, MA, United States), Phospho-Akt (Ser473) (D9E) XP (#4060, 1:2000, rabbit, Cell Signaling), S6 ribosomal protein (5G10) (#2217, rabbit, 1:1000, Cell Signaling), Phospho-S6 ribosomal protein (ser240/244) (#2215, 1:1000, rabbit, Cell signaling), p38 (M138) (mouse, abcam, #ab31828, 1:1000), Phospho-p38 MAPK(Thr180/Tyr182) (rabbit, Cell Signaling, 9211, 1:1000), Collagen Type I (#14695-1-AP, 1:1000, rabbit, Proteintech, Rosemount, IL, United States), Collagen Type III (N-terminal) (#22734-1-AP, 1:500, rabbit, Proteintech), GSK3β (#22104-1-AP, 1:1000, rabbit, Proteintech), and Phospho-GSK3β (ser9) (#67558-1-Ig, 1:2000, mouse, Proteintech). After washing three times with TBS-T, membranes were incubated for 1 h at room temperature with the following secondary antibodies: Goat anti-mouse IgG Cy3 (Cytiva, #PA43009, 1:2500), Goat anti-rabbit IgG Cy5 (#PA45011, 1:2500, Cytiva), or HRP-conjugated Goat anti-Mouse IgG (#31430, 1:10,000, Thermo Fisher Scientific) and Goat anti-Rabbit IgG (#31460, 1:10,000, Thermo Fisher Scientific). Proteins were visualized using the SuperSignal West Pico PLUS Chemiluminescent substrate (#34577, Thermo Fisher Scientific) and imaged with a Bio-Rad ChemiDoc system. Molecular weights were estimated using PageRuler Plus Prestained Protein Marker (#26619, Thermo Fisher Scientific) or ECL Plex Fluorescent Rainbow Markers (#RPN851E, Cytiva). For SDC4, as a loading control, the total protein was used (#92–11021, Revert 700 Total Protein Stain, LI-COR Biosciences, Lincoln, NE, United States).

### 2.3 Immunocytochemistry

Cells were cultured on sterilized glass coverglass (#631-1577, VWR, Radnor, PA, United States) in a 6-well plate. Following experimental treatments, the media were removed, and cells were washed twice with phosphate-buffered saline (PBS). Fixation was carried out using 4% paraformaldehyde (PFA) in PBS for 10 min at room temperature, followed by a single wash with PBS. Permeabilization was achieved by incubating the cells in 0.1% Triton X-100 in PBS for 15 min. Cells were incubated with 1× Blocking Buffer (ab126587, Abcam) in PBS for 1 h at room temperature to block non-specific binding. Primary antibody incubation was performed overnight at 4 °C with antibodies diluted in 0.1× Blocking Buffer. The primary antibodies included Syndecan-4 (rabbit, Genscript, 1:1000) and HA-tag (mouse, #32–6700, 1:250, Thermo Fisher Scientific). After overnight incubation, cells were washed three times with PBS-T (PBS with 0.1% Tween-20) before incubating with fluorophore-conjugated secondary antibodies in the dark for 1 h at room temperature. Secondary antibodies used were Goat anti-mouse Alexa Fluor 488 (#A10667, 1:400, Thermo Fisher Scientific) and Goat anti-rabbit Alexa Fluor 546 (#A11010, 1:400, Thermo Fisher Scientific). For nuclear staining, NucBlue (#R37605, Thermo Fisher Scientific, MA, United States) was added to the secondary antibody solution at one drop per 500 µL. Following the final PBS-T wash, the glass coverslips with cells were mounted on slides using DAKO Fluorescent Mounting Media (S302380-2, Agilent, Santa Clara, CA, United States). Imaging was conducted with fluorescence microscopy analysis (ZEISS Axio Observer Z1 microscope, Jena, Germany) with ×10 objective, and images were processed using Adobe Photoshop CS3.

### 2.4 RNA isolation and RT-qPCR

Total RNA was isolated using the RNeasy mini kit (#74104, Qiagen, Germantown, MD, United States) according to the manufacturer’s instructions, including DNase treatment. Cells were washed twice by cold PBS, and cDNA was generated from 300 ng or 1 µg using LunaScript RT SuperMix kit (#E3010L, New England BioLabs, Ipswich, MA, United States) in a 20 µL reaction volume with random hexamers, according to the manufacturer’s instructions. The cDNA was diluted in RNAse-free water and 10 ng cDNA was used in the following RT-qPCR reactions performed using Luna Universal probe RT-qPCR Master Mix (#M3004X, New England BioLabs, MA, United States) and the QuantStudio 5 PCR System (Applied Biosystems, Waltham, MA, United States). The amplification protocol consisted of an initial denaturation step at 95 °C for 1 min, followed by 40 cycles of denaturation at 95 °C for 15 s, and extension at 60 °C for 30 s. Gene expression of the samples was normalized against the EEF2 reference gene. A comparison of the relative gene expression between control and treated cells was derived by using the comparative Ct method. In short, ΔΔCt values were obtained by subtracting the average ΔCt mean of control samples (i.e., lipofectamine or solvents for blocking peptides) from the ΔCt of the treated samples, and the relative gene expression was then calculated by the formula 2^−ΔΔCt^. The statistical calculations were performed using the ΔCt and ΔΔCt values. All TaqMan® primers and probes are listed in Table 1. The RT-qPCR was performed in technical triplicates on at least three independent experiments.

**TABLE 1:** Gene target and TaqMan®primer/probe assays.

Gene target	TaqMan®primer/probe assays (#4351372)
*EEF2*	Gg03339740_m1
*TGFb1*	Gg07156069_g1
*IL1b*	Gg03347157_g1
*ACTA2*	Gg03352404_m1
*COL1A1*	Gg07167955_g1
*COL3A1*	Gg03325764_m1
*SDC1*	Gg07175697_s1
*SDC2*	Gg03345644_m1
*SDC3*	Gg03339851_m1
*SDC4*	Gg03370419_m1
*DCN*	Gg03355063_m1
*MMP2*	Gg03365277_m1
*MMP9*	Gg03338324_g1

### 2.5 Statistical analysis and software

Data are shown as mean ± SEM (standard error of the mean). All quantifications of protein expression from Western blots (n = 3–6) were quantified by ImageQuantTL 10.2–499 (Cytiva, GE Healthcare Life Sciences, Marlborough, MA, United States) with the background subtracting method of the rolling ball (radius 2). For RT-qPCR (n = 3–7) and immunoblotting (n = 3–6), an unpaired two-tailed t-test or one-way ANOVA with Brown-Forsythe and/or Welch correction were used. The statistical analyses were performed in Graph Pad Prism version 10.4.0 (GraphPadSoftware, La Jolla, CA, United States). As indicated in each figure, statistical significance was considered with P-values <0.05. Jalview 2.11.4.0 was used for the alignment of SDC4 sequences.

## 3 Results

### 3.1 SDC4 overexpression reduces fibrotic markers

**FIGURE 1:**
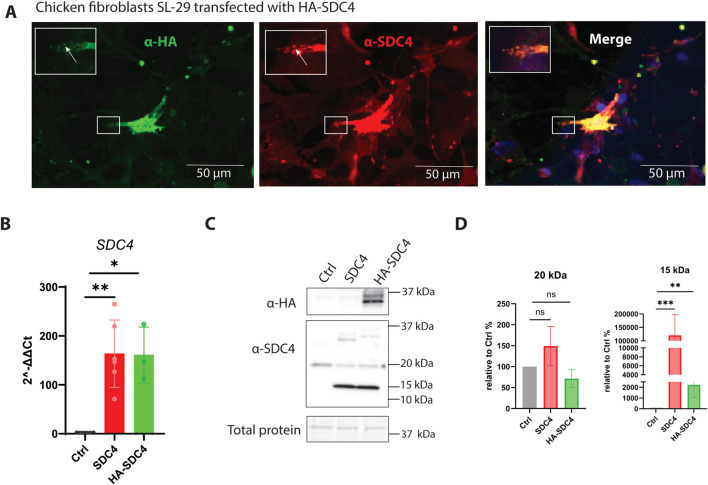
Overexpression of SDC4 in SL-29 chicken fibroblast cells. **(A)** Chicken embryonic SL-29 fibroblasts were transiently transfected with HA-tagged SDC4, fixed with 4% PFA, stained with mouse α-HA (left) and rabbit α-SDC4 (middle) followed by goat anti-mouse Alexa Fluor 488 and goat anti-rabbit Alexa Fluor 546 before fluorescence microscopy analyses. NucBlue was used to stain nuclei (blue). The inserts represent a higher magnification of the framed areas. Arrows indicate focal adhesions. Scalebar 50 µm. **(B)** SL-29 cells were transiently transfected with SDC4 (n = 6), or HA-SDC4 (n = 3) plasmids. Lipofectamine only was used as control (Ctrl). Bars show the relative mRNA gene expression in transfected cells compared with the mean average of Ctrl cells (n = 6). The bars are presented as ± SEM. Asterisks denote significant differences between Ctrl and transfected cells, statistics assessed using unpaired t-test with Welch correction (ns > 0.05; *p ≤ 0.05; **p ≤ 0.01). **(C)** A representative western blot showing the level of SDC4 after transient transfection of the chicken fibroblasts. Cells treated as in B were subjected to western blotting using antibodies against HA tag adding a molecular size increase around nine amino acid (top) or only the cytoplasmic part of SDC4 (middle panel). The lowermost panel shows total protein. **(D)** Quantification of the levels of the 15 and 20 kDa SDC4 fragments, normalized to total protein level. The bars represent n = 8 in one technical replicate for SDC4 and Ctrl, and n = 4 in one technical replicate for HA-SDC4. The bars are presented as ± SEM. Asterisks denote significant differences between Ctrl and transfected cells, statistics assessed using unpaired Mann-Whitney non-parametric t-test (ns > 0.05; **p ≤ 0.01; ***p ≤ 0.001).

To investigate the cellular localization of SDC4 in embryonic chicken fibroblasts, Hemagglutinin (HA)-tagged SDC4 plasmid was transiently transfected into SL-29 cells and visualized with an antibody against the N-terminal HA-tag, or with a custom-made antibody recognizing the C-terminal of SDC4, in a fluorescence microscope. Consistent with the literature (Baciu and Goetinck, 1995; Rønning et al., 2015), SDC4 localized to the cytoplasmic membrane and presumably focal adhesions (Figure 1A). RT-qPCR confirmed the high gene expression of *SDC4* using plasmids with and without the HA-tag, and no difference in relative gene expression was observed in cells transfected with these two plasmids (Figure 1B). We did not see any significant effects using an empty vector (Supplementary Figure S1), suggesting the impact of overexpression observed was not an artifact of transfection with plasmid. Upon investigating SDC4 protein expression after overexpression, we detected bands of approximately 35 kDa, corresponding to the full-length SDC4/HA-SDC4 proteins. These bands were either identified using antibodies against the HA-tag (Figure 1C, top box), located in the N-terminus of the extracellular domain of SDC4, or against the cytoplasmic region of SDC4 (Figure 1C, lower box). We also observed a 15 kDa SDC4 fragment in cells overexpressing *SDC4*, as a remaining fragment of SDC4 after shedding (Figures 1C,D). A 20 kDa fragment was observed independently of *SDC4* overexpression, suggesting additional regulatory mechanisms for SDC4 shedding.

**FIGURE 2:**
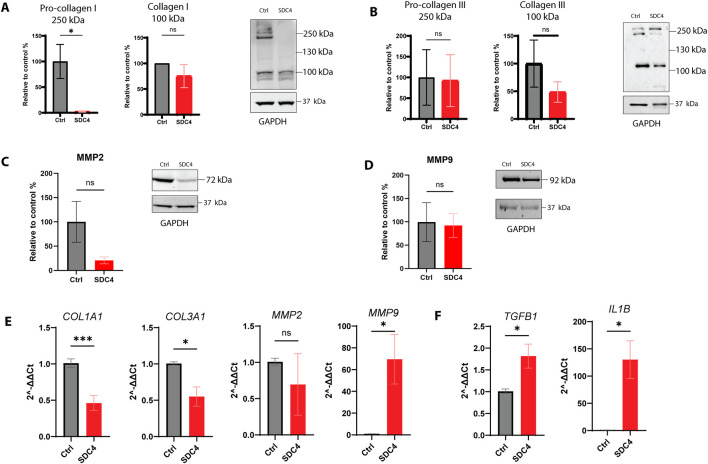
Effect of SDC4 overexpression on fibrosis markers in chicken fibroblasts SL-29. Protein expression of **(A)** pro-collagen I beta chain (250 kDa) and alpha chain (100 kDa), **(B)** pro-collagen III (250 kDa) and collagen I (100 kDa), **(C)** MMP-2 and **(D)** MMP-9 in SDC4 transfected SL-29 cells. GAPDH was used as a reference protein. The bars represent n = 3 in one technical replicate. Gene expression (n = 6 in technical triplicates) of **(E)** COL1A1, COL3A1, MMP2, MMP9, **(F)** TGFβ and IL1β in SDC4 transfected SL-29 cells. The bars are presented as ± SEM. Asterisks denote significant differences between Ctrl (lipofectamine only) and transfected cells, statistics assessed using unpaired t-test with Welch correction and Mann-Whitney U (ns > 0.05; *p ≤ 0.05; **p ≤ 0.001).

*SDC4* overexpression did not affect the relative gene expression of any other SDC family members (*SDC1-3*) (Supplementary Figure S2). On the other hand, the overexpression of *SDC4* had a notable impact on the protein (Figures 2A–D) and relative gene expression of several fibrotic markers (Figure 2E), including a decrease in gene expression of *COL1A1* and *COL3A1* and an increase in *MMP9*. No significant changes in the mRNA level were detected for *MMP2*. The protein expression of fibrosis markers, such as pro-collagen I beta bands (250 kDa) was significantly decreased after *SDC4* overexpression (Figure 2A). No significant changes were observed in the protein expression of the alpha bands (100 kDa) of collagen I (Figure 2A) and both alfa and beta forms of collagen III (Figure 2B) (Amirrah et al., 2022). Compared to gene expression MMP9 and MMP2 (Figure 2E) showed a decreasing tendency in *SDC4*-overexpressing fibroblasts (Figure 2E). Moreover, cytokines such as transforming growth factor beta-1 (*TGFB1*) and interleukin-1 beta (*IL1B*) showed a substantial increase in gene expression (Figure 2F), suggesting that *SDC4* overexpression triggers inflammatory responses. Additionally, the protein expression of α-smooth muscle actin (α-SMA, *ACTA2*) (Supplementary Figure S3A) and also its detection at the gene expression level (Supplementary Figure S3B) suggest that the SL-29 cells already displayed a myofiber phenotype in the culture system, and *SDC4* overexpression did not influence this further. Finally, the relative gene expression of *DCN* (Supplementary Figure S3C) was unchanged.

To summarize, *SDC4* overexpression in embryonic chicken fibroblasts reduced the protein and gene expression of fibrosis markers, such as collagens and MMP-2 and MMP-9, while enhancing the expression of inflammatory cytokines *TGFB1* and *IL1B*. Additionally, overexpression led to the shedding of SDC4, with no significant changes in the relative gene expression of other syndecan family members.

### 3.2 *SDC4* overexpression influences MAPK signaling, but not Wnt/β-catenin and Akt signaling pathways

**FIGURE 3:**
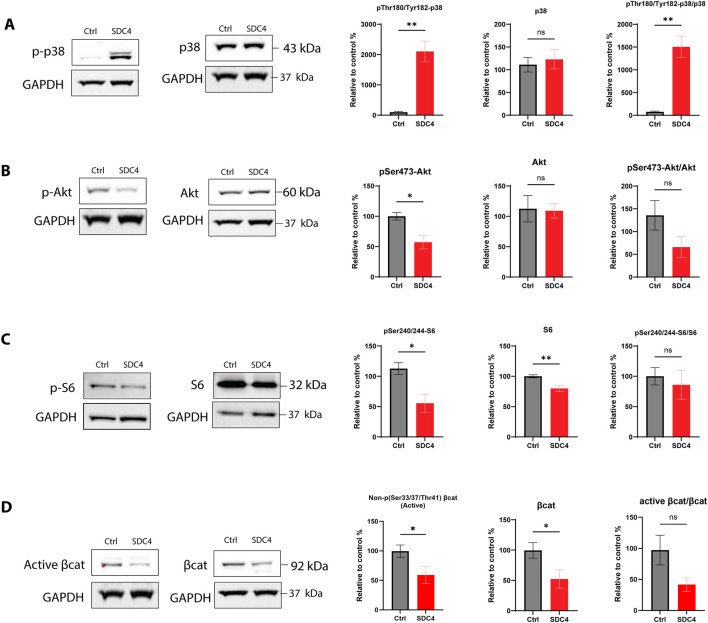
Effect of SDC4 overexpression on signaling pathways chicken fibroblasts. Protein expression analysis following SDC4 overexpression in SL-29 chicken fibroblasts. Detected proteins include **(A)** phosphorylated p38 at Thr186/Tyr182 and total p38, **(B)** phosphorylated Akt at Ser473 (pSer473- Akt) and total Akt, **(C)** phosphorylated ribosomal protein S6 at Ser240/244 (pSer240/244-S6) and total ribosomal protein S6, and **(D)** non-phosphorylated (active) β-catenin at Ser33/37 and Thr41 and total β-catenin. The ratio of phosphorylated to total or active to total protein was calculated for each protein, illustrating the impact of SDC4 overexpression. As a control (ctrl) was used treatment with lipofectamine only. GAPDH was used as a reference protein. The bars are presented as ± SEM from n = 3-6 in one technical replicate. Asterisks denote significant differences between Ctrl and transfected cells, statistics assessed using either unpaired t-test with Welch correction or Mann-Whitney U test (ns > 0.05; *p ≤ 0.05; **p ≤ 0.01).

To explore the potential effect of SDC4 on signaling pathways associated with fibrosis, such as MAPK, Wnt/β-catenin, and Akt, we investigated their protein expression in cells transiently overexpressed with SDC4. We could see a dramatic increase in p38 activation (phosphorylation at Thr180/Tyr182) and in the p-p38/p38 ratio, while the total p38 protein expression was unchanged (Figure 3A). Akt activation (Ser473 phosphorylation) was significantly decreased, but total Akt and their ratio showed only reducing tendency (Figure 3B). The ribosomal protein S6, a downstream target of Akt, consistently showed reduced Ser240/244 phosphorylation and total S6 protein (Figure 3C). Protein β-catenin active and non-active (phosphorylated) showed downregulation with a decreasing tendency in their ratio (Figure 3D). Finally, ERK1/2 and its phosphorylated (Thr202/Tyr204) version appeared not affected by *SDC4* overexpression (Supplementary Figure S4A), nor was GSK3β (Supplementary Figure S4B).

*SDC4* overexpression enhanced p38 MAPK phosphorylation, a pathway involved in ECM synthesis and pro-fibrotic responses. At the same time, *SDC4* overexpression decreased Akt activation (phosphorylation), and β-catenin levels, with no apparent effect on ERK1/2 or GSK3β, suggesting a modulation of fibrotic signaling pathways.

### 3.3 Effect of blocking peptides on fibrosis and syndecan-4 shedding

**FIGURE 4:**
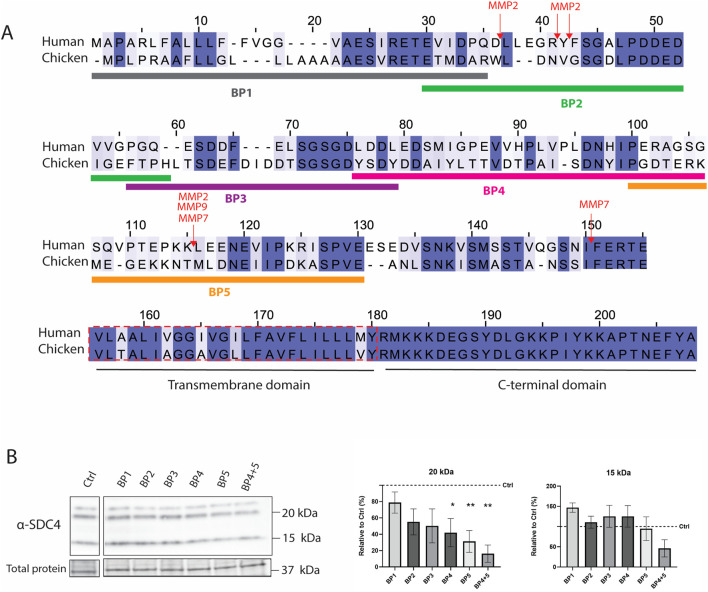
Effect of blocking peptides on SDC4 shedding. **(A)** Alignment of human and chicken SDC4 protein sequences, highlighting amino acid percentage identity (dark blue >80%, lighter shades of blue are >60% and >40%, white <40%). Five overlapping blocking peptides (BP1-5) representing the SDC4 ectodomain were designed and are marked by different colors below the chicken SDC4 sequence. Red arrows indicate MMP cleavage sites in human SDC4 (Manon-Jensen et al., 2013). Alignment created by Jalview 2.11.4.0. **(B)** Effect of BP1-5 on SDC4 shedding in SDC4-overexpressing chicken fibroblasts (N = 4 in one technical replicate). Total protein is used as a loading control. The bars on the right are presented as ± SEM. SDC4-overexpressing fibroblasts treated with the solvent of BP serve as the control (Ctrl, stippled line). Asterisks denote significant differences between Ctrl and transfected cells, statistics assessed using either unpaired t-test with Welch correction or Mann-Whitney U test (*p ≤ 0.05; **p ≤ 0.01).

**FIGURE 5:**
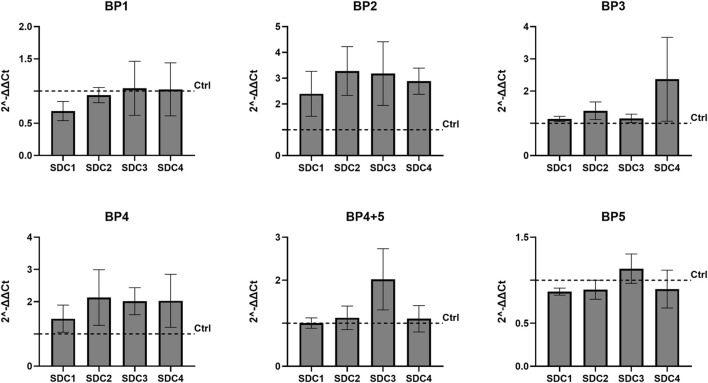
Effect of blocking peptides on the gene expression of the various SDCs. Effect of BP1-5 on the levels of SDC1-4 expression in SDC4 transfected chicken fibroblasts SL-29 showing no significant differences compared to control (Ctrl). All results were compared to the appropriate solvent for each blocking peptide. Gene expression was measured in three biological replicates, each performed in technical triplicates, and statistical significance was calculated using one-way ANOVA with Welch and Brown-Forsythe corrections. The bars are presented as ± SEM.

**FIGURE 6:**
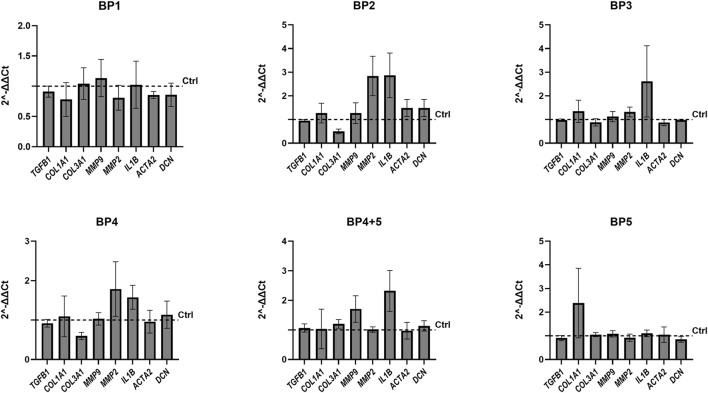
Effect of blocking peptides on the gene expression of fibrosis markers. Effect of BP1-5 on TGFB1, COL1A1, COL3A1, MMP9, MMP2, IL1B, ACTA2, DCN gene expression levels in SDC4 transfected chicken fibroblasts SL-29. All results were compared to the appropriate solvent for each blocking peptide. Gene expression was measured in three biological replicates, each performed in technical triplicates, and statistical significance was calculated using one-way ANOVA with Welch and Brown-Forsythe corrections. The bars are presented as ± SEM.

To investigate if shedding had any effect on fibrotic markers, five overlapping blocking peptides (BP1-5) representing the chicken SDC4 ectodomain were designed (Figure 4A). Notably, the corresponding human sequences of BP2 and BP5 have been described to contain several MMP cleavage sites (Manon-Jensen et al., 2013) (Figure 4A). We have previously shown that BP5, containing AA100-130 of chicken SDC4, inhibits shedding in chicken skeletal muscle satellite cells, reducing the appearance of the 15 kDa SDC4 fragment (Pejšková et al., 2024). Adding the BP4 and BP5 to the chicken fibroblast cells overexpressing *SDC4* showed a reduction in the 20 kDa fragment (Figure 4B). A higher band, approximately 22 kDa, was detectable only in some experiments (data not quantified and shown) and may correspond to the full-length chicken SDC4 core protein, excluding GAGs, approximately 21.5 kDa. No significant decrease in expression of the 15 kDa cellular fragment was apparent in contrast to the results in the muscle satellite cells (Pejšková et al., 2024), indicating cell-specific effects. None of the blocking peptides influenced the relative gene expression of any members of the SDC family (Figure 5), nor did they significantly affect the relative gene expression of any of the fibrotic markers investigated (Figure 6). However, some trends were observed: BP2 treatment, which did not affect shedding (Figure 4B), and BP4 which did, exhibited both a decreasing trend in *COL3A1* gene expression, while showing a trend for increased *MMP2* and *IL1B* expression (Figure 6). Effect of the BP1-5 solvents (controls) on gene expression are shown in Supplementary Figure S5, with only minor impact of DMSO observed for *COL3A1* and *TGFB1* and a significant anomaly noted in the water solvent control for *MMP9*. In summary, blocking peptides BP4 and BP5 reduced the 20 kDa SDC4 fragment in fibroblasts, with BP4 also showing trends in decreasing *COL3A1* expression and increasing *MMP2* and *IL1B*, while overall expression of SDCs and fibrotic markers remained largely unchanged.

### 3.4 TGF-β1 affects syndecan-4 shedding in chicken fibrosis

**FIGURE 7:**
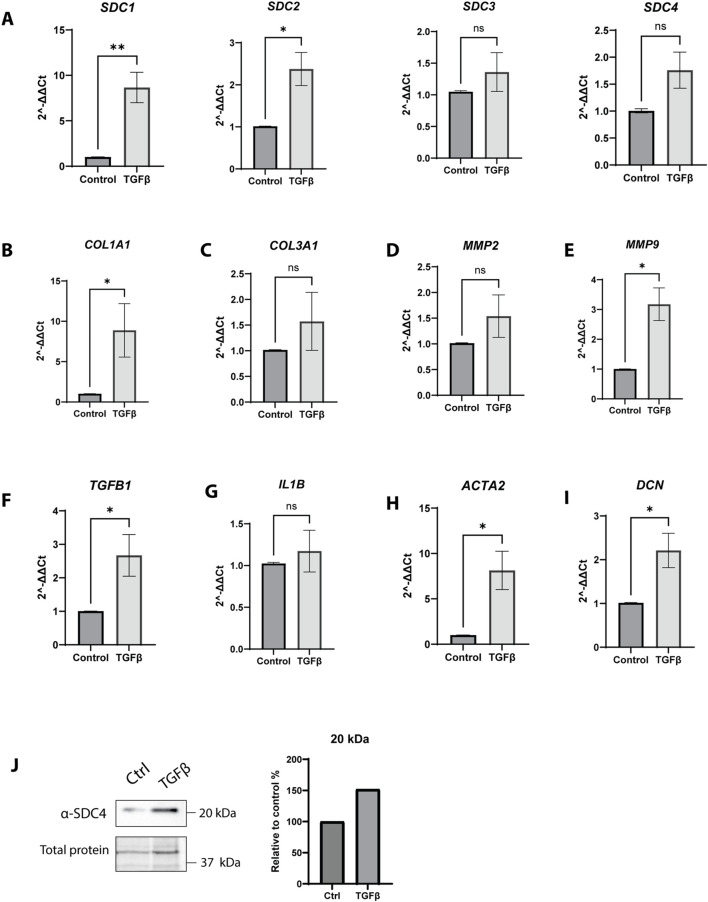
Effect of TGF-β1 on different gene and protein expression levels. Chicken fibroblasts were treated with TGF-β1 for 24 h. The gene expression levels were assessed for **(A)** syndecans (SDC1-4) and various fibrotic markers, including **(B)** COL1A1, **(C)** COL3A1, **(D)** MMP2, and **(E)** MMP9. Additionally, cytokines such as **(F)** TGFB1 and **(G)** IL1B were measured, and the expression of **(H)** ACTA2, a myofibroblast marker, and **(I)** DCN, a small leucine-rich proteoglycan. Results are expressed as fold change relative to the control (Ctrl; SL-29 cells treated with the solvent of TGF-β1). Gene expression (n = 7, in technical triplicates) was calculated by an unpaired t-test with Welch’s correction. ns > 0.05; *p ≤ 0.05; **p ≤ 0.01. The bars are presented as ± SEM. **(J)** Immunoblotting analysis of the 20 kDa SDC4 fragment following 24-h treatment with TGF-β1, compared to control (Ctrl) conditions. Quantification (N = 3 in one technical replicate) is presented as the mean, normalized to total protein, and expressed as a percentage relative to the control.

*SDC4* overexpression resulted in a significant increase in gene expression of cytokines like *TGFB1* or *IL1B* (Figure 2). Therefore, chicken fibroblasts were treated with TGF-β1 for 24 h to investigate its effect on SDC1-4 gene expression and SDC4 shedding, as well as gene expression of fibrotic markers. TGF-β1 stimulation increased gene transcriptions of *SDC1* and -*2,* and with tendency for SDC4 but not of *SDC3* (Figure 7A). The effect of TGF-β1 on fibrotic markers showed an increase in expression of *COL1A1* and *MMP9* but not *COL3A1* or *MMP2* (Figures 7B–E). *TGFB1*, *ACTA2*, and *DCN* were all increased by TGF-β1 treatment (Figures 7F,H,I), but *IL1B* remained unchanged (Figure 7G). Interestingly, western blot analysis showed increased appearance of the 20 kDa form of SDC4 protein after 24h TGF-β1 treatment (Figure 7J). We also performed an initial study with different cytokines, including IL-1β, IL6, LPS, and TNF-α, which in contrast to TGF-β1 showed non-significant effects on the mRNA levels of *SDCs* (Supplementary Figure S6). In summary, *SDC4* overexpression and TGF-β1 treatment promote increase in *TGFB1* gene expression, whereas only TGF-β1 stimulation induces a clear pro-fibrotic response and *SDC1* and *SCD2* at gene level and increase SDC4 shedding (20 kDa).

## 4 Discussion

### 4.1 Syndecan-4 has anti- and pro-fibrotic effects in chicken fibroblasts

Skeletal muscle myopathies, such as wooden breast (WB) myopathy in chicken, are characterized by fibrosis, which is a hallmark of disease progression and contributes to impairment of the muscle function (Sihvo et al., 2014; Velleman et al., 2017). SDC4 has previously been shown to be key modulator in reducing lung fibrosis during inflammatory conditions (Jiang et al., 2010; Tanino et al., 2019), but also a trigger of pro-fibrotic processes in cardiac tissue (Herum et al., 2013; Lunde et al., 2022). In this study, we investigated the role of SDC4 and its shedding in regulating fibrosis, and its interactions with TGF-β1 in chicken fibroblasts. Our results showed that *SDC4* overexpression both promotes anti- and pro-fibrotic responses, suggesting this to be linked to increasing shedding and upregulation of cytokines. SDC4 stimulated *TGFB1* gene expression, *versus* TGF-β1 stimulated increases shedding of SDC4 highlighting a complex regulatory pathway between SDC4 and TGF-β1 in fibrosis.

Fibrosis is typically characterized by various fibrotic markers, such as collagens and matrix metalloproteinases (MMPs), contributing to ECM remodeling. Tanino et al. (2019) demonstrated that SDC4 knockout in lung fibroblasts increases collagen I and III mRNA and protein content, reflecting an anti-fibrotic role of SDC4. This aligns with our findings regarding the SDC4 overexpression model, where we observed a decrease in COL1A1 and COL3A1 mRNA and pro-collagen I protein production. On the contrary, Herum et al. (2013) demonstrated in cardiac fibroblast from SDC4 KO mice a decrease in *Col1a1* and *Col3a1* expression. Increased levels of pro-collagen III (dos Santos Moreira et al., 2015) and pro-collagen I have been suggested as *in vivo* markers of cardiac fibrosis in mice (Bouffard et al., 2008). Moreover, *SDC4* overexpression reduced MMP-2 protein expression, as well as a trend toward decreasing MMP-9 protein expression. However, in our study, *MMP9* mRNA levels increased, which might show different regulation of MMPs on gene and protein level, as we also reported in our earlier study (Pejšková et al., 2023). The increase in *MMP9* gene expression can be linked to the elevated cytokine *IL1B* expression (this study), which is identified as an inducer of MMP-9 (Roderfeld et al., 2006).

Decorin has previously been found as a suppressor of TGF-β1-induced fibrosis (Wang et al., 2023) and has been proposed as anti-fibrotic protein in epidural fibrosis (Ding et al., 2021). Decorin has also been shown to have a role as regulator of collagen crosslinking (Weber et al., 1996) and as a potential regulator of myofibroblast activation (Klingberg et al., 2013). However, no effect of *SDC4* overexpression on decorin (*DCN*) gene expression was demonstrated in our study (Supplementary Figure S3C). Similarly, α-SMA (*ACTA2*), a well-established marker of myofibroblasts, was observed both at the gene and protein level of our SL-29 cells, regardless of *SDC4* overexpression (Supplementary Figure S3A,B), suggesting that this cell line already inherited myofibroblast characters. Supporting our observation, the SDC4 knockout mouse model showed no changes in α-SMA, but in contrast to our data, no changes in *COL1A1* and *COL3A1* mRNA expression in the later stages of pressure-overload cardiac fibrosis (Lunde et al., 2016).

In contrast to the anti-fibrotic effect of SDC4 suggested discussed above, *SDC4* overexpression also increased pro-fibrotic markers such as *TGFB1* and *IL1B*. Elevated levels of IL-1β and TNF-α are well-documented in heart fibrosis (Yndestad et al., 2007), and lipopolysaccharide (LPS), a pro-inflammatory component of gram-negative bacterial cell walls, has been shown to increase *SDC4* expression in cardiac fibroblasts and myoblasts (Strand et al., 2013). Nevertheless, in our study, treatment of fibroblasts with cytokines such as IL-1β, TNF-α, IL-6, or LPS for 24 h did not alter the gene expression of *SDC4* or other SDCs (Supplementary Figure S6). In endothelial cells, exposure to LPS or IL-1β for 4 h led to decreased expression of SDC1 and SDC2, while SDC4 expression increased it (Vuong et al., 2015). By 24 h, these expression levels returned to baseline or exhibited only minor changes (Vuong et al., 2015). This transient response may explain our lack of observed changes within the experimental timeframe.

TGF-β, has been reported to play a major role in modulating fibrosis via SDC4 signaling in the lungs (Tanino et al., 2019). In our study, TGF-β1 treatment of chicken fibroblasts did not alter SDC4 gene expression, despite the upregulation of other well-known fibrosis markers (*COL1A1, MMP9, ACTA2,* and *DCN*). Interestingly, an effect was observed on *SDC1* and *SDC2*. This suggests that SDC4 induces *TGFB1* production, but TGF-β1 does not regulate SDC4 expression at the gene level.

### 4.2 Syndecan-4 shedding in *SDC4*-overexpressing fibroblasts

Our earlier research has demonstrated increased shedding of all SDCs in WB myopathy (Pejšková et al., 2023; Pejšková et al., 2024). In chicken fibroblasts overexpressed with SDC4, *SDC4* increased dramatically together with an observed produced 15 kDa SDC4 fragment, additionally to 20 kDa fragment present regardless of SDC4 gene expression. This observation was similar to the muscle satellite cells from affected and normal chicken with observation of additional decreasing 10 kDa SDC4 fragment in affected samples (Pejšková et al., 2024). These results show that the shedding pattern differs in the fibroblasts and the muscle satellite cells, both in SDC4-dependent and independent manners.

The shedding of SDCs is a critical regulatory mechanism in inflammation and is known to increase in response to cytokines, including TGF-β, IL-1β, and TNF-α (Marshall et al., 2003; Strand et al., 2013; Vuong et al., 2015; Li et al., 2016). In our case, TGF-β1 stimulation significantly increased shedding of the 20 kDa SDC4 fragment, although SDC4 gene expression remained without significant changes. This observation suggests that TGF-β1 may influence SDC4 shedding by another mechanism than increase expression of SDC4. Strand et al. (2013) demonstrated that the ectodomain of SDC4, resulting from increased shedding in diseased hearts, generate soluble pro-inflammatory molecules that activate immune cell recruitment pathways and propagate local immune responses within cardiac tissue. In our study, *SDC4* overexpression increased mRNA levels of cytokines such as *TGFB1* and *IL1B*, supporting a dual role of SDC4 in triggering inflammation additionally to reducing fibrosis. Lunde et al. (2016) suggested that full-length SDC4 has pro-fibrotic properties by increasing collagen levels in cardiac fibroblasts, whereas shed SDC4 fragments exhibit anti-fibrotic effects. However, our observation of increased inflammation markers and SDC4 shedding induced by TGF-β1 supports the idea that the pro-fibrotic effect is driven by increased shedding, contrast with the previous study.

### 4.3 Syndecan-4 modulates p38 MAPK and Akt signaling

Fibrosis involves multiple signaling pathways, many of which intersect with SDC4 signaling (Astudillo et al., 2014; Foglia et al., 2019; Qin et al., 2021; Støle et al., 2022). This study investigated well-characterized pathways such as MAPK, Akt, and Wnt/β-catenin, focusing on their modulation following *SDC4* overexpression in chicken fibroblasts.

Our findings demonstrated that overexpression of *SDC4* resulted in enhanced activation of p38 MAPK. Previous studies have indicated that p38 MAPK plays a role in the production of pro-inflammatory and pro-fibrotic mediators (Sugiyama et al., 2012) and participates in ECM synthesis (Lee et al., 2019), suggesting that SDC4 may support pro-fibrotic effects in chicken fibroblasts. On the other hand, while our earlier research demonstrated ERK1/2 upregulation in WB myopathy *in vivo* (Pejšková et al., 2023), no changes were detected in WB-affected muscle satellite cells (Pejšková et al., 2024), and, similarly, *SDC4* overexpression did not significantly impact ERK1/2 levels in chicken fibroblasts in this study. This demonstrates that the MAPK pathway, particularly through ERK1/2, plays a crucial role in WB myopathy, while p38 MAPK can positively influence fibrosis via SDC4; however, the specific contributions of these pathways may vary depending on the cellular context and the specific regulatory mechanisms involved during extracellular matrix remodeling.

The role of Akt signaling in fibrosis has been well-established, particularly in cardiac fibrosis (Qin et al., 2021). Our study demonstrated that *SDC4* overexpression in chicken fibroblasts led to decreased Akt activation. In line with our findings, female SDC4-knockout mice exhibited increased Akt activation in skeletal muscle and cardiomyocytes (Rønning et al., 2020; Støle et al., 2022). Contrary, reduced mTORC2 activity in SDC4-null endothelial cells has been shown to diminish Akt activation (Partovian et al., 2008), further supporting the view that SDC4 modulates Akt signaling through complex and tissue and cell-dependent pathways. Akt can regulate fibrosis primarily through its ability to phosphorylate and inactivate GSK3β at Ser9 (Hardt and Sadoshima, 2002; Zheng et al., 2020). However, in our study, pSer9-GSK3β levels remained unchanged, indicating that SDC4 may regulate fibrotic processes downstream of Akt through alternative mechanisms or proteins. Interestingly, ribosomal protein S6, a downstream target of Akt involved in regulating protein synthesis and cellular growth, was similarly downregulated in fibroblasts overexpressing *SDC4*. This finding is consistent with *in vivo* observations in WB myopathy, where ribosomal protein S6 and its activation were decreased (Pejšková et al., 2023).

SDC4 is a major regulator of cellular response to Wnt signals by facilitating the induction of the canonical Wnt signaling pathway (Astudillo et al., 2014; Clarke et al., 2015). It has been reported that suppressing the Akt/GSK3β/β-catenin signaling pathway ameliorates pulmonary fibrosis (Liu et al., 2021). We have also shown that β-catenin increased its active form during WB myopathy (Pejšková et al., 2023). In this study, the downstream mediator of the canonical Wnt signaling pathway, β-catenin, and its active form were downregulated after *SDC4* overexpression. β-catenin is usually regulated by the upstream molecule GSK3β, which is required for phosphorylation and proteasomal ubiquitination of β-catenin (Guo et al., 2017), however, as mentioned above, we did not observe any changes GSK3β.

### 4.4 Blocking of syndecan-4 shedding

The precise cleavage sites in the SDC4 ectodomain remains insufficiently characterized. Previous studies have shown that deletion of the SDC4 ectodomain (Δ138-145) or mutations in its GAG attachment sites (S44A, S62A, S64A) significantly reduce SDC4 shedding (Strand et al., 2013). Furthermore, MMPs, which are key enzymes responsible for syndecan shedding have been reported to reduce the amount of shed SDC4 *in vitro* when MMP-9 is inhibited or knocked down (Bollmann et al., 2021). We hypothesized that blocking peptides (BPs) derived from the SDC4 ectodomain might bind to MMPs, thereby reducing SDC4 shedding and modulating fibrotic responses. In our study, BPs, specifically BP4 and BP5, demonstrated significant reductions in shedding of 20 kDa SDC4 core protein fragment in *SDC4* overexpressing chicken fibroblasts, however with no impact on the gene expression of fibrotic markers. This suggests that this shedding activity in the cell is not associated with collagen production, although further research is needed. Intriguingly, differences in SDC4 shedding patterns across cell types are observed. Our previous research in muscle satellite cells revealed that BP5 reduced shedding of the SDC4 core protein (15 kDa) (Pejšková et al., 2024). However, after treatment with BPs, there were no significant changes in the shedding of the 15 kDa remaining SDC4 core protein in chicken fibroblasts. Manon-Jensen et al. (2013) identified cleavage sites for MMP-2 and MMP-9 on human SDC4 at K105, a site replaced by threonine in chicken SDC4. BP5 was designed to represent this region, and the significant decrease we observed in the 20 kDa SDC4 fragment could be directly linked to it. Further studies are required to identify if shedding activity producing 15 kDa SDC4 fragment plays a regulatory role in fibrosis.

## 5 Conclusion

In conclusion, our study shows both anti-fibrotic and pro-fibrotic effects of SDC4 overexpressed in chicken fibroblasts. *SDC4* overexpression decreased most of the fibrosis markers on gene and protein level. On the other hand, *SDC4*-overexpressing fibroblasts exhibits pro-fibrotic effects, likely through ectodomain shedding and modulation of p38 MAPK and Akt signaling pathways. Elevated gene expression of pro-inflammatory cytokines such as *TGFB1* and *IL1B* suggests that SDC4 also is involved in initiating inflammatory responses in chicken fibroblasts. The distinct shedding patterns of SDC4 in chicken fibroblasts and muscle satellite cells (Pejšková et al., 2024) highlight the complexity of its regulatory mechanisms, with MMPs playing a crucial role. BPs effectively reduced SDC4 shedding but did not affect gene expression of SDC4 or fibrotic markers. Further research is needed to fully elucidate the mechanisms underlying SDC4 shedding and its impact on fibrosis, which could pave the way for novel treatments for skeletal muscle myopathies and other fibrotic diseases.

## Data Availability

The original contributions presented in the study are included in the article/Supplementary Material, further inquiries can be directed to the corresponding authors.
