# Supplementary material for: Dual roles of syndecan-4 in regulating chicken fibrosis in vitro
Source: Front Physiol. 2026 Mar 23;17:1782914. doi: 10.3389/fphys.2026.1782914 (PMC13050698; doi:10.3389/fphys.2026.1782914)
Supplement: Supplementary file 1 [file Supplementaryfile1.docx]

***Supplementary Material***


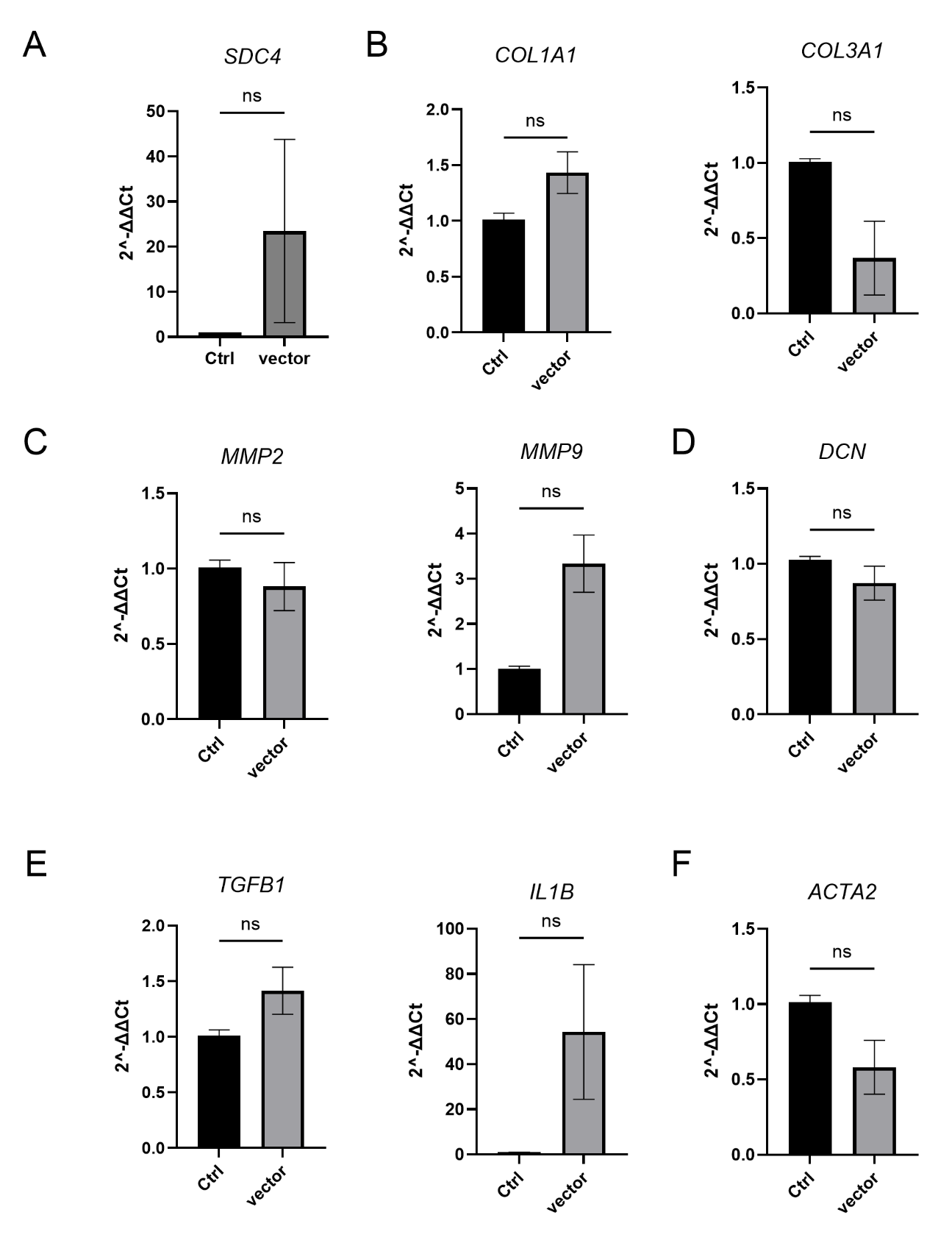


***Figure S1. Vector effect in SDC4-overexpressing chicken fibroblasts.*** *Influcence of transfection of empty vector (pCEP4) (n=3 in technical triplicates) into chicken fibroblasts SL-29 compared to control (Ctrl, lipofectamine only) (N=6) on* ***(A)*** *SDC4,* ***(B)*** *collagens - COL1A1 and COL3A1,* ***(C)*** *sheddases – MMP2 and MMP9,* ***(D)*** *DCN,* ***(E)*** *cytokines - TGFB1 and IL1B, and* ***(F)*** *ACTA2. Individual experiments were measured in technical triplicates. The bars are presented as ± SEM. Significant differences were detected using un-paired t-test with Welsh correction (ns>0.05).*


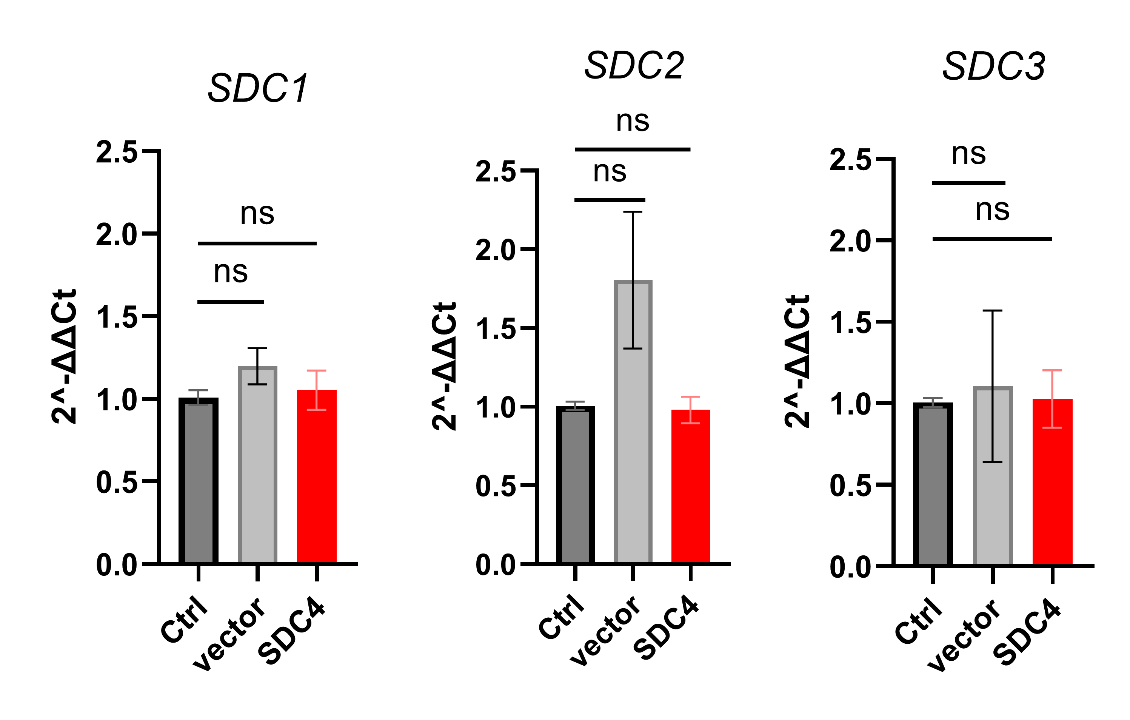


***Figure S2. Investigation of SDC1 to -3 mRNA level after SDC4 overexpression in chicken fibroblasts.*** *Embryonic chicken fibroblasts SL-29 were transfected with SDC4 (N=6) or empty vector (pCEP4) (N=3) and compared to control (Ctrl, lipofectamine only) (N=6 in technical triplicates). The bars are presented as ± SEM. Significant differences were detected using un-paired t-test with Welsh correction (ns>0.05).*


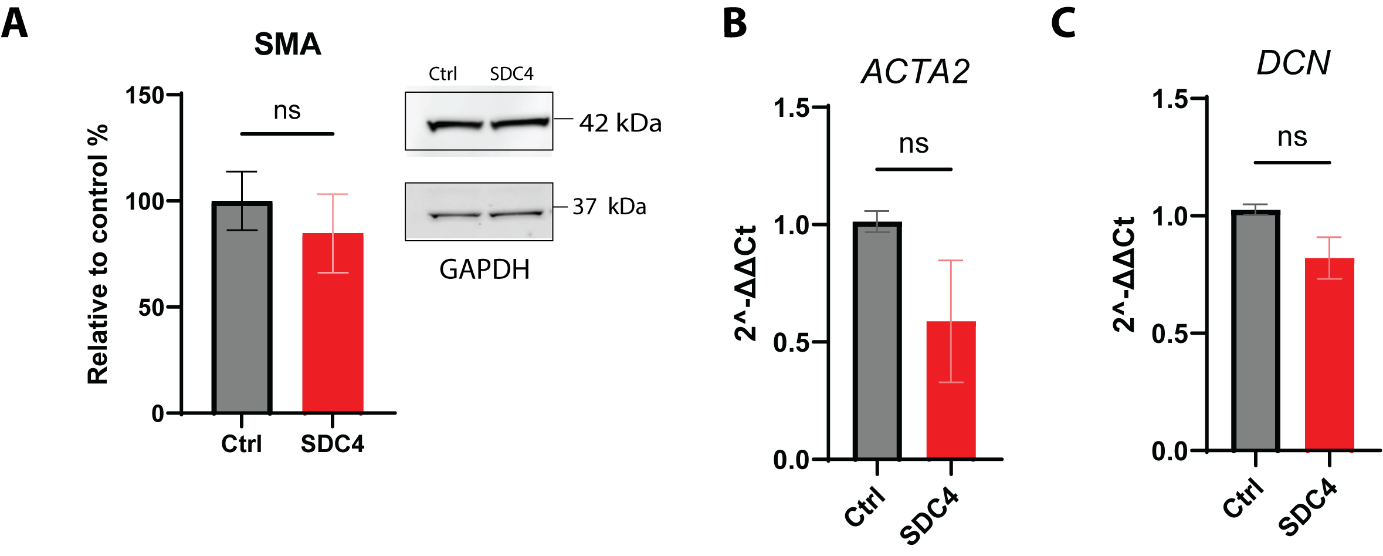


***Figure S3. Protein and/or gene expressions of SMA (ACTA2) and DCN in SDC4 transfected chicken fibroblasts SL-29.*** ***(A)*** *SMA protein expression* *measured by immunoblotting (N=4 in one technical replicate). Lipofectamine only was used as negative control (ctrl). Data are normalized to GAPDH and presented as persentage relative to control (ctrl) with SEM. Gene expression of* ***(B)*** *ACTA2 and* ***(C)*** *DCN measured by RT-qPCR. Gene expression data (N=6 in technical triplicates) show fold change to control (Ctrl). Individual experiments were measured in technical triplicates. GAPDH was used as a loading control, and protein levels were normalized accordingly. The bars are presented as ± SEM. Significant differences between groups were detected using unpaired t-test with Welsh correction (ns>0.05).*


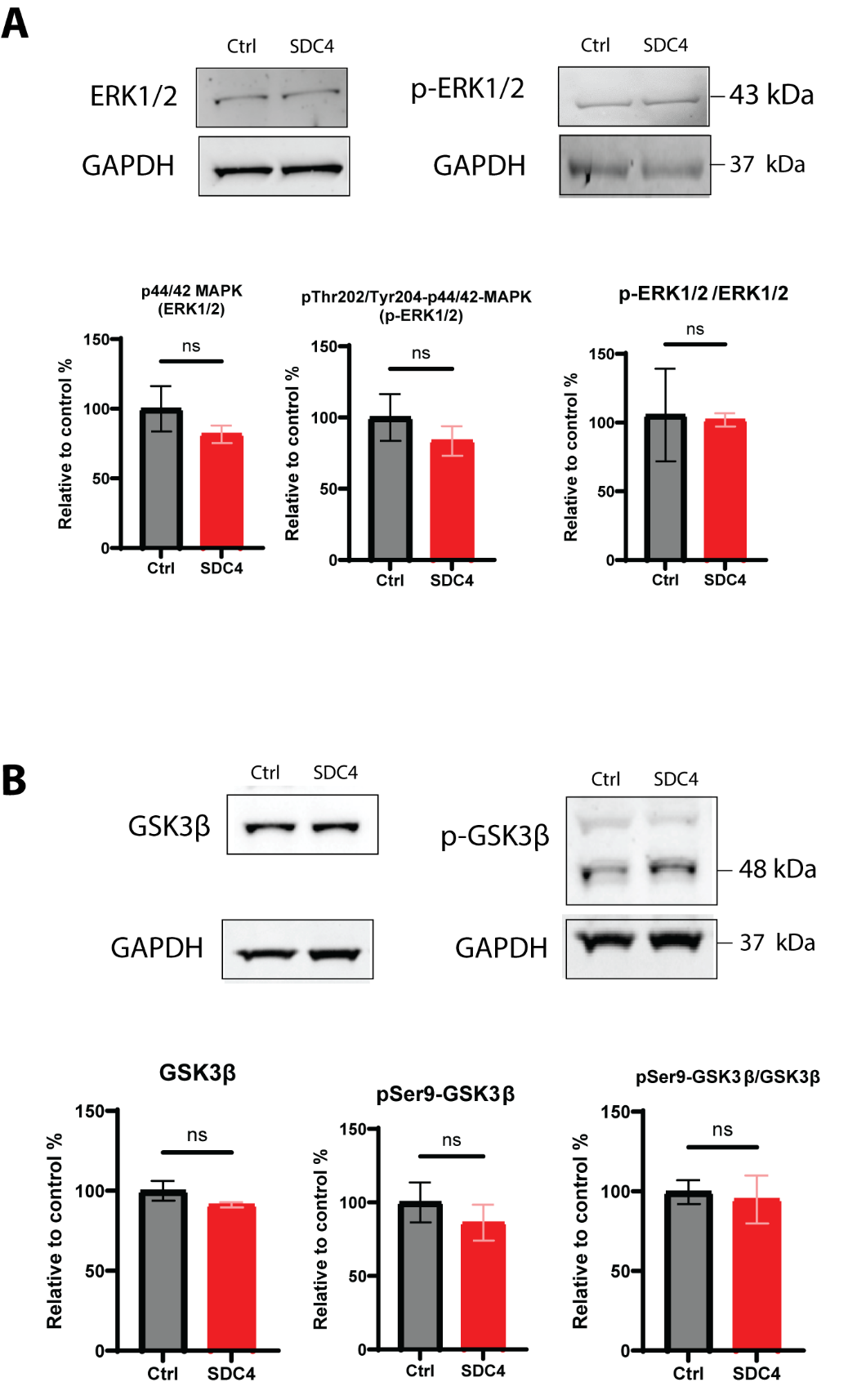


***Figure S4. Effect of SDC4 overexpression on ERK1/2 and GSK3β levels.*** *Chicken fibroblasts SL-29 were transfected with SDC4 or lipofectamine only (ctrl). Immunoblotting of* ***(A)*** *ERK1/2 and pThr202/Tyr204 ERK1/2 (shown as p-ERK1/2) and* ***(B)*** *GSK3β and pSer9-GSK3β after SDC4 transfection. GAPDH was used as a loading control, and protein levels were normalized accordingly. The bars are presented as ± SEM. Significant differences (N=2 in one technical replicate) were detected using unpaired t-test with Welsh correction (ns>0.05).*


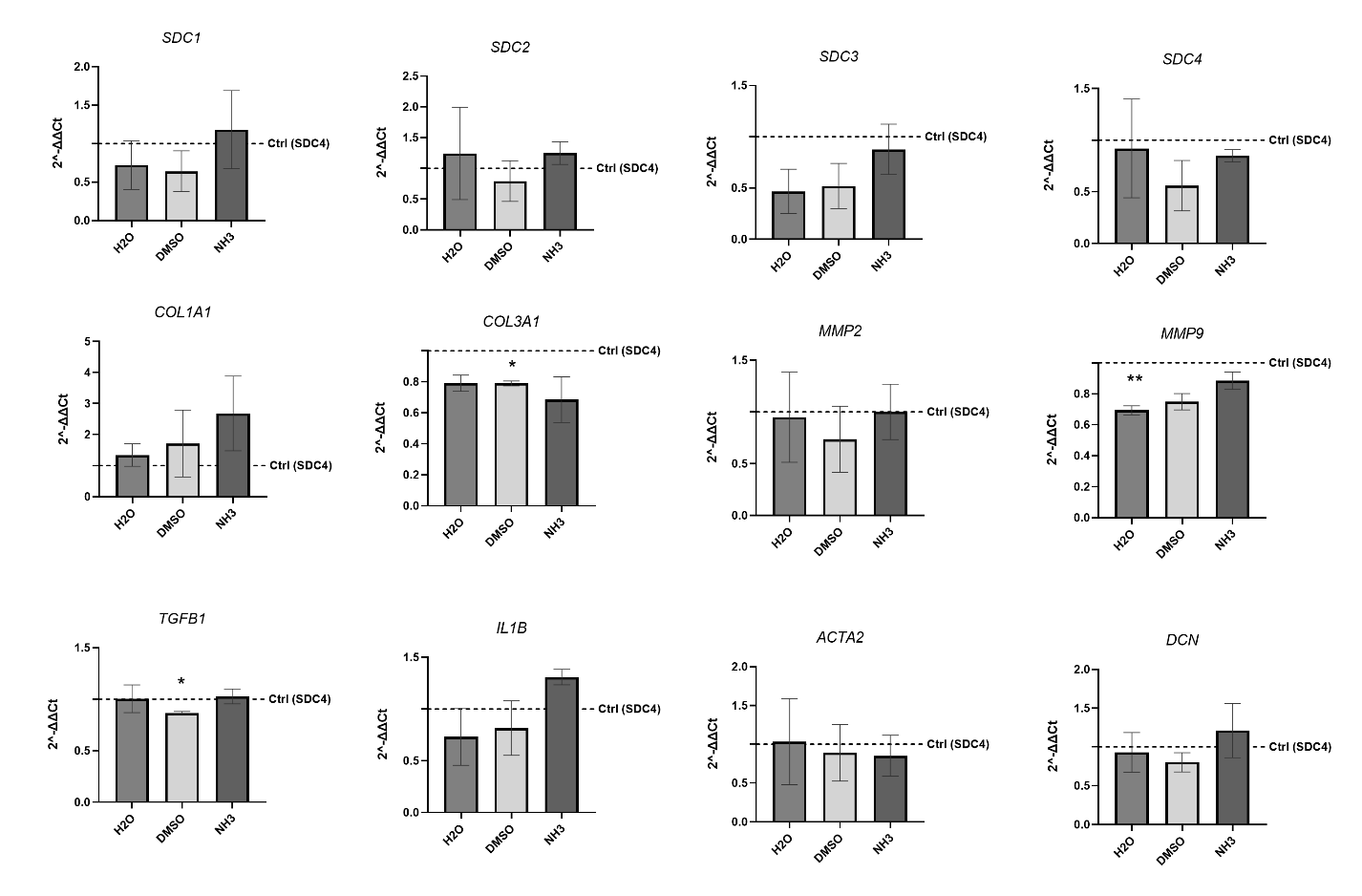


***Figure S5. Blocking peptides solvent controls.*** *Different solvents were used for different blocking peptides. Every blocking peptide was normalized to its own solvent control and presented as Ctrl in the main figure 6. To observe difference, all data were normalized to SDC4 overexpression (Ctrl SDC4). DMSO effect was observed in case of gene expression of COL3A1 and TGFB1. Gene MMP9 was significantly affected by H_2_O . The remaining genes examined in this study did not exhibit any significant changes in expression due to the solvent. Significant differences (N=3 in technical triplicates) were detected using one-way ANOVA with Brown-Forsythe and Welsh correction.*


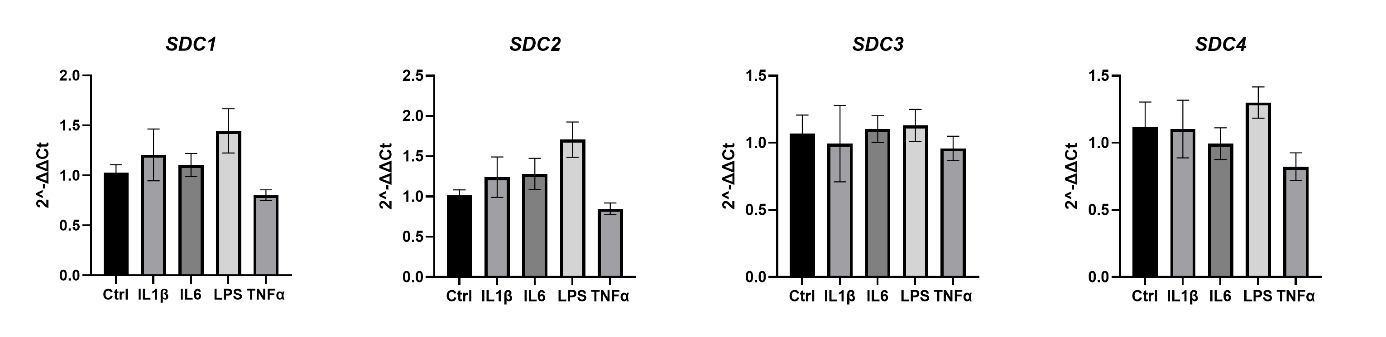


***Figure S6. Expression of SDC genes after 24h with cytokine treatments.*** *Chicken fibroblasts SL-29 were stimulated for 24h with cytokines (IL-1β, IL-6, LPS, TNF-α) and gene expression of SDCs was measured. Individual experiments (N=3) were measured in technical triplicates. The bars are presented as ± SEM. Significant differences were detected using one-way ANOVA with Brown-Forsythe and Welsh correction.*

**Supplementary non-cropped WB**

**
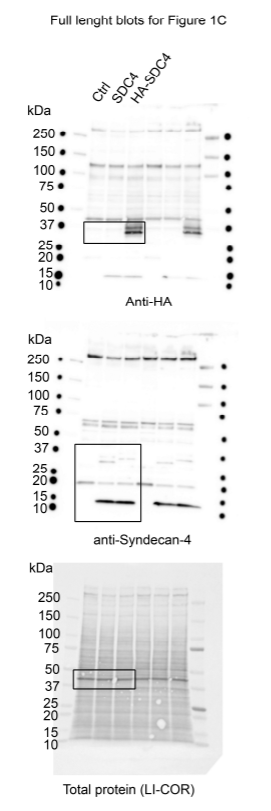
**

**Full length blot for Figure 2B**

**
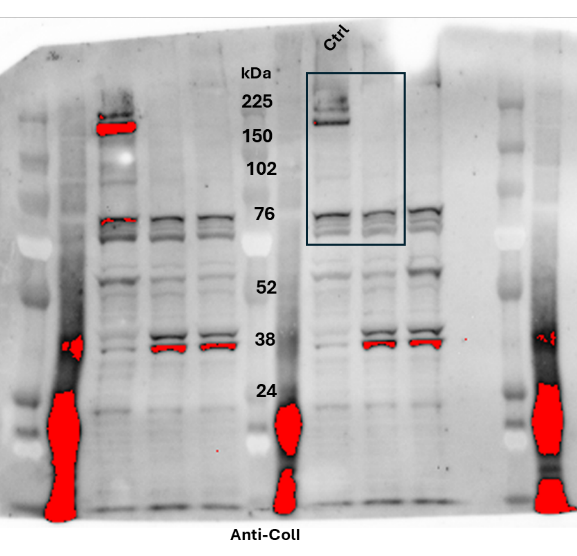

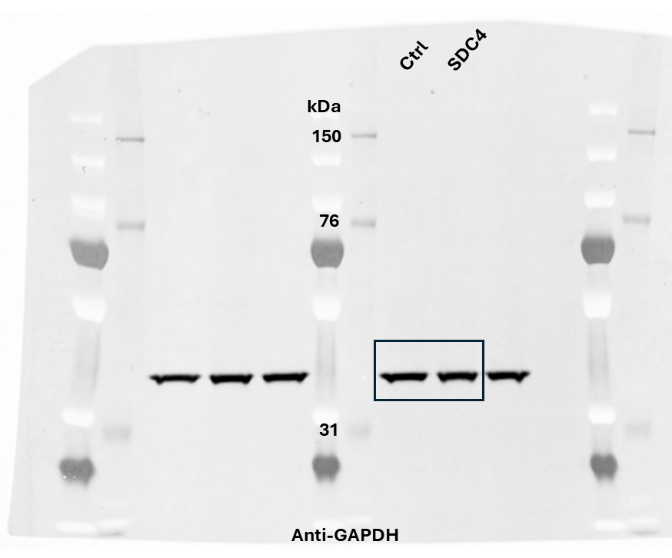
**

**Full length blot for Figure 2C**

**
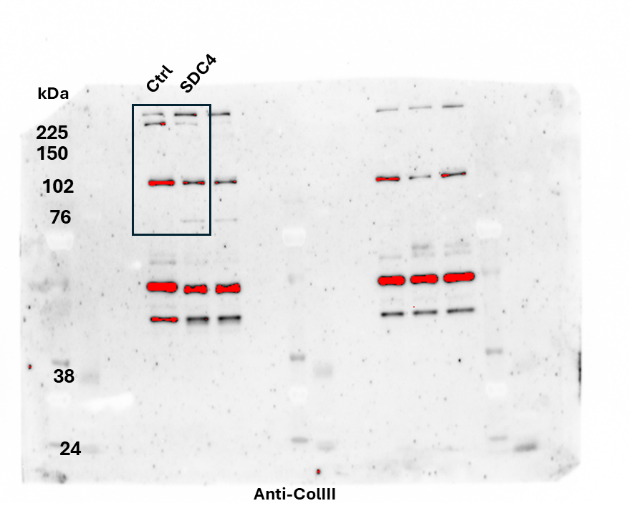

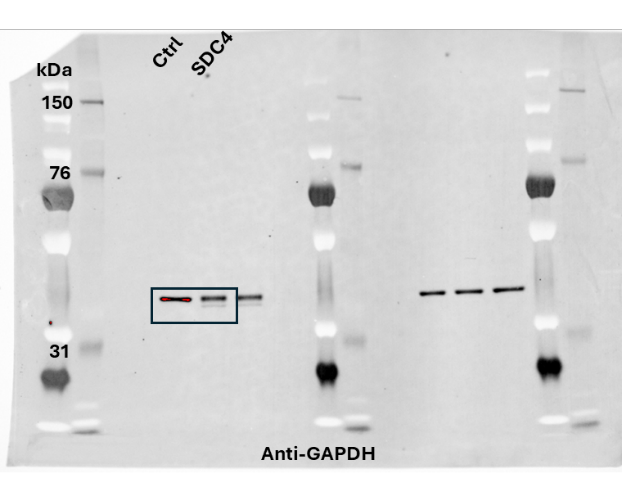
**

**Full length blot for Figure 2D**

**
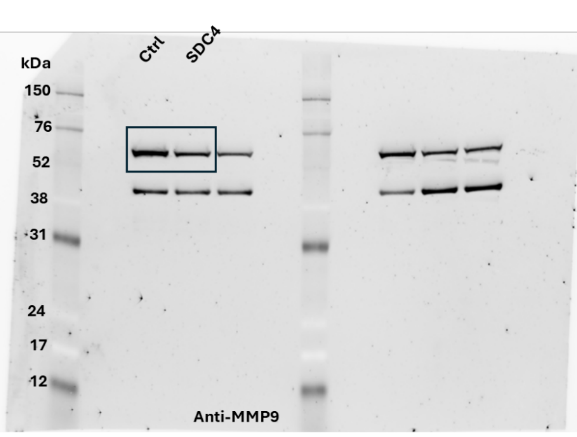

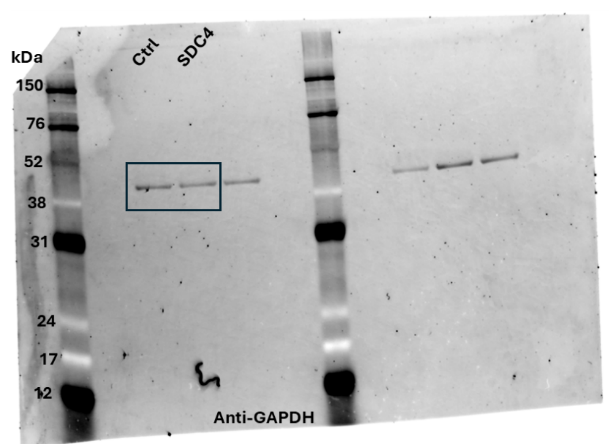
**

**Full length blot for Figure 2E**

**
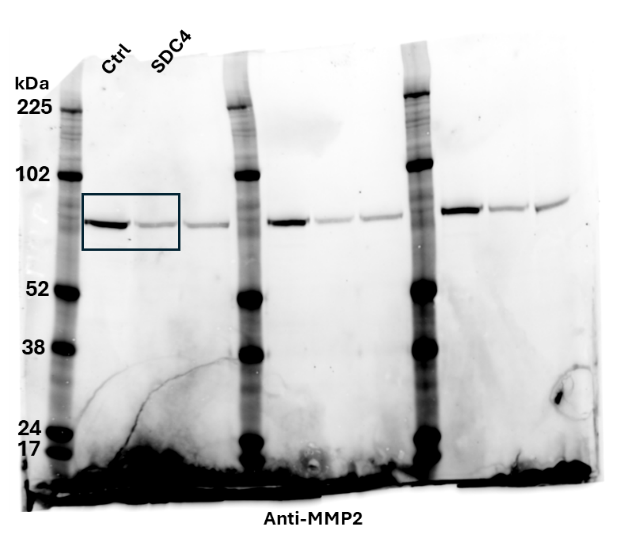

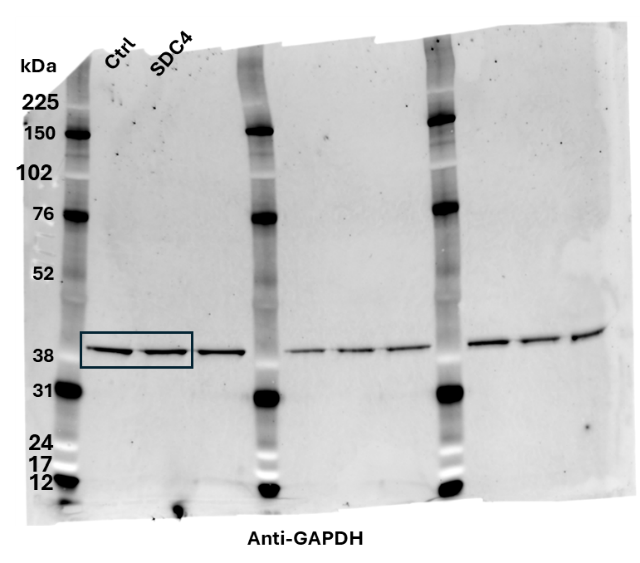
**

**Full length blot for Figure 3A**

**
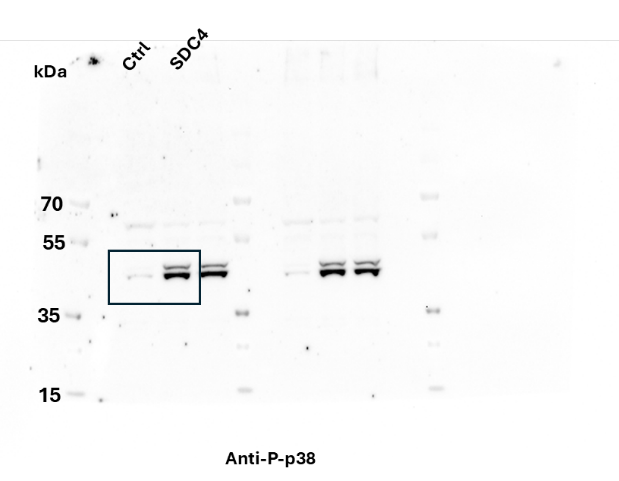

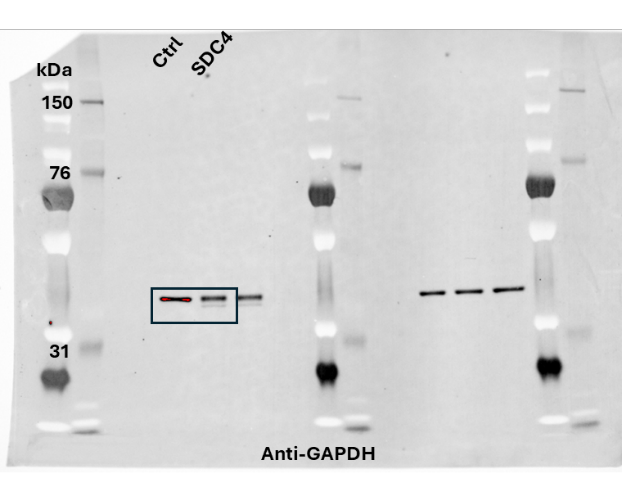
**

**
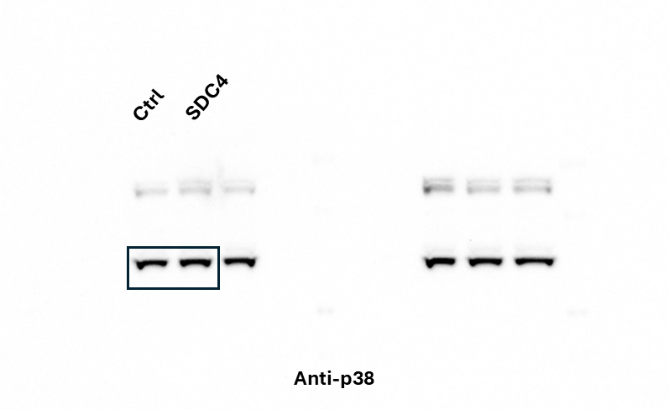

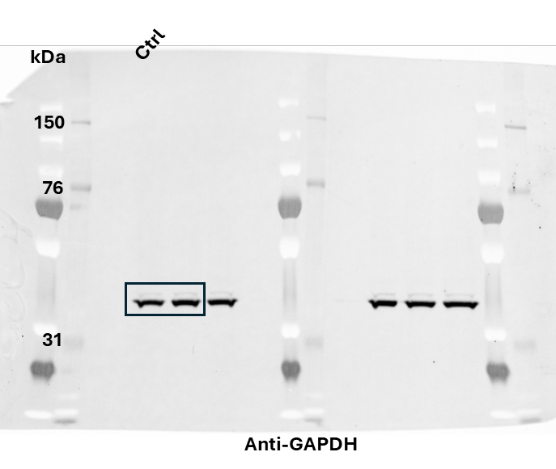
**

**Full length blot for Figure 3B**

**
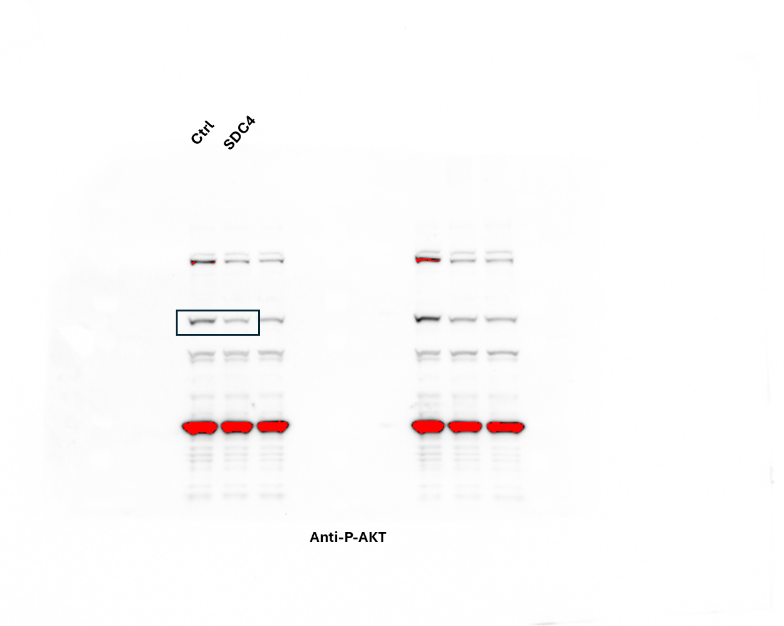

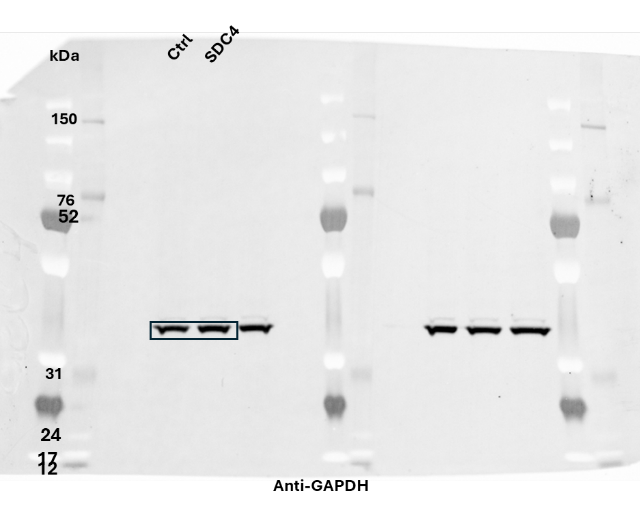
**

**
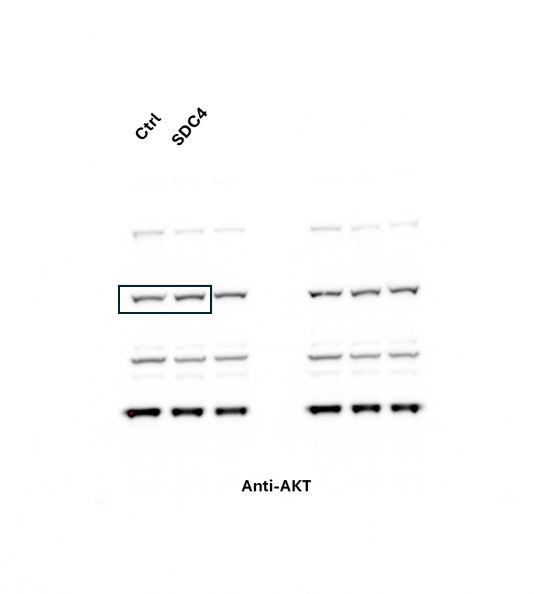

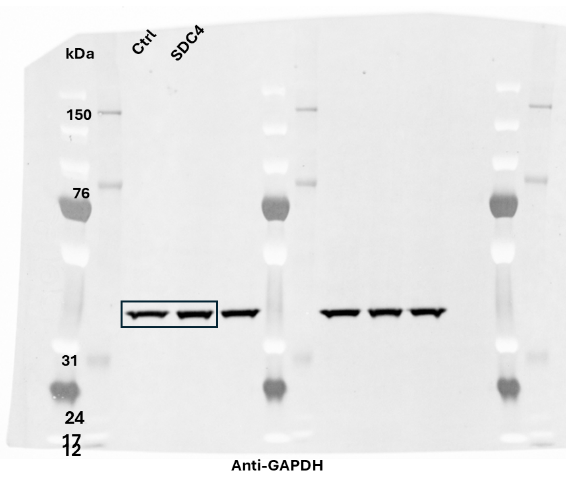
**

**Full length blot for Figure 3C**

**
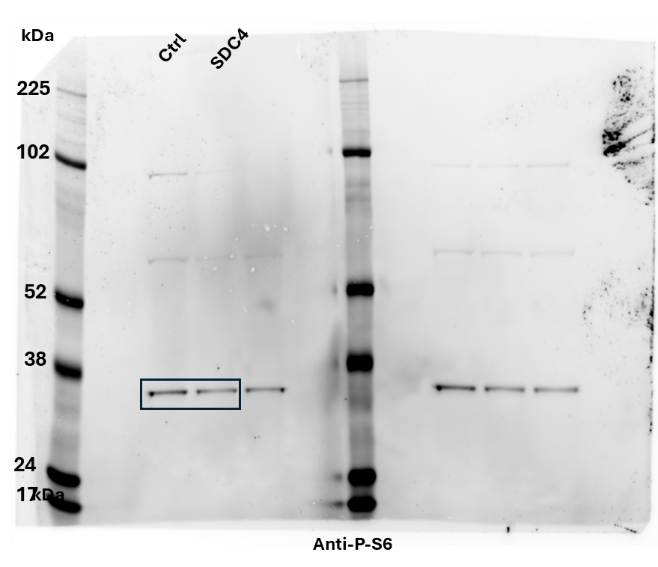

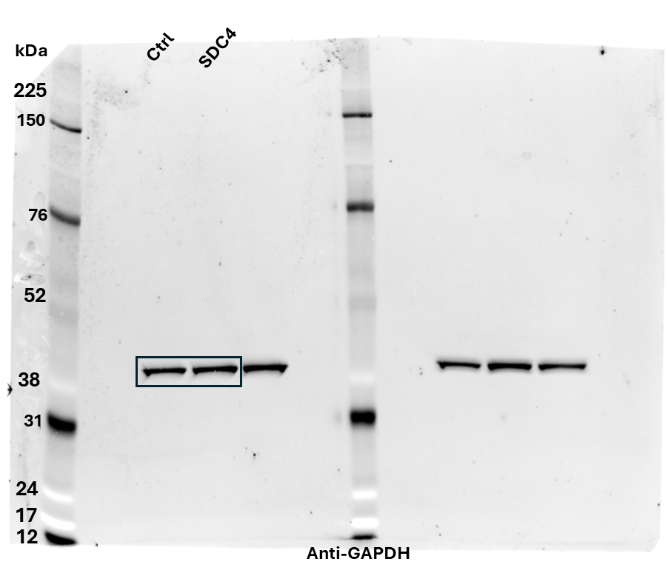
**

**
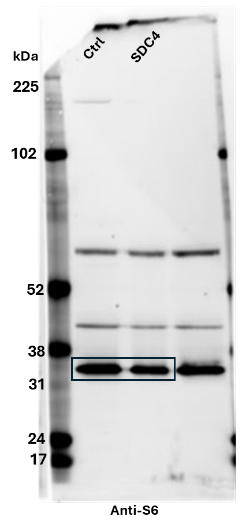

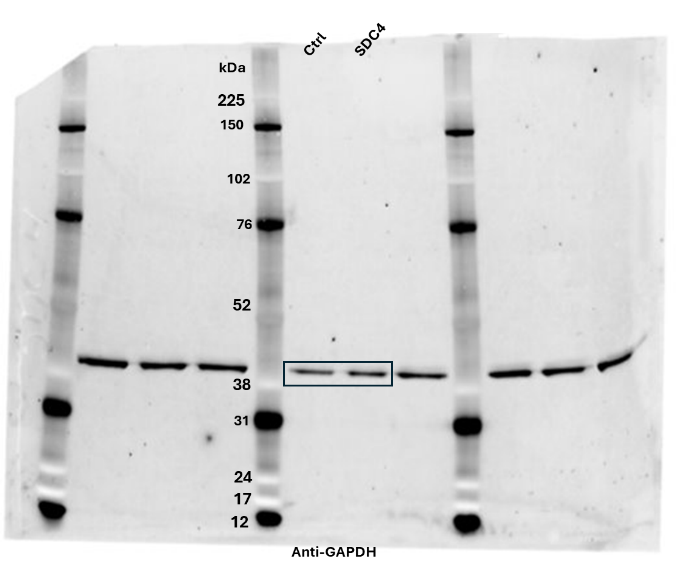
**

**Full length blot for Figure 3D**

**
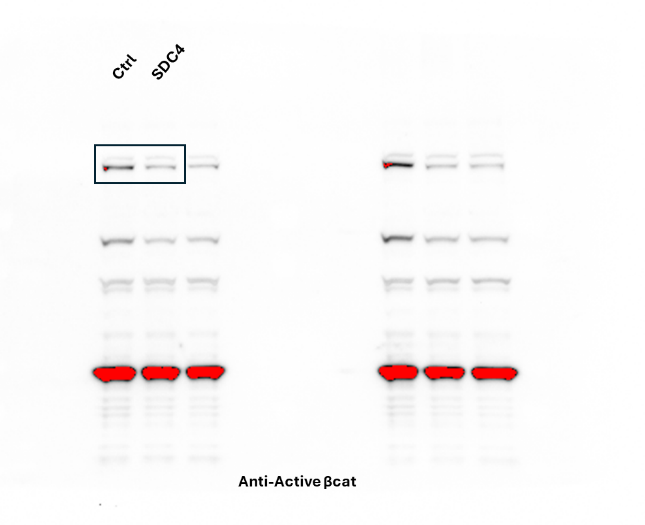

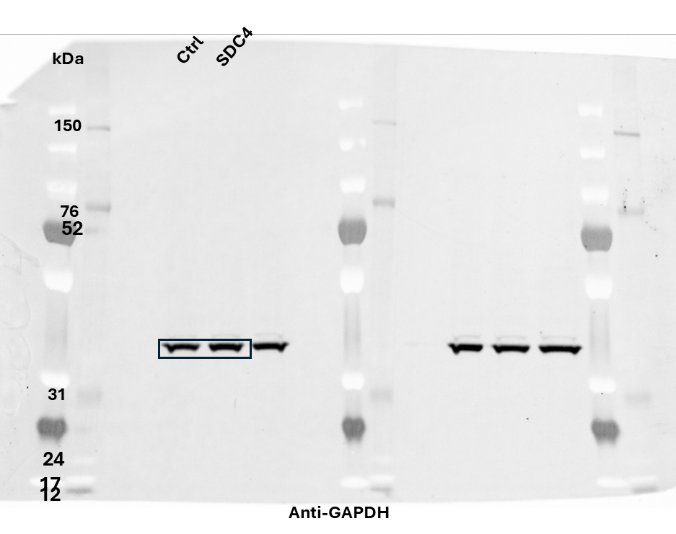
**

**
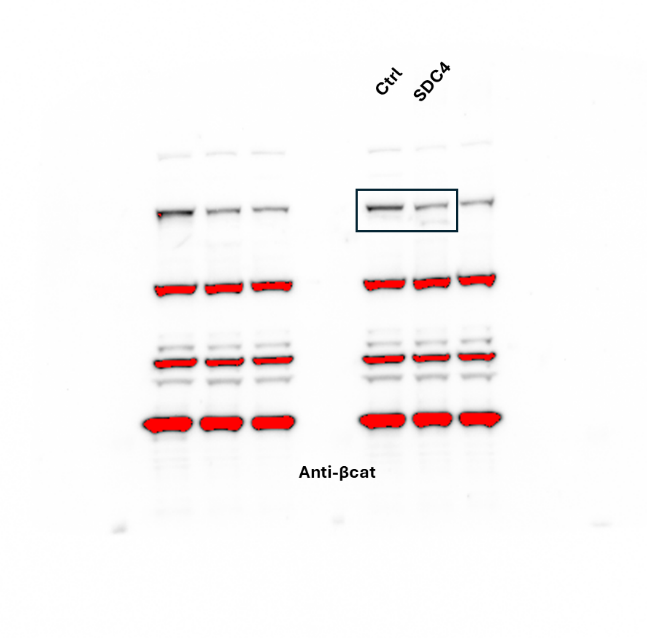

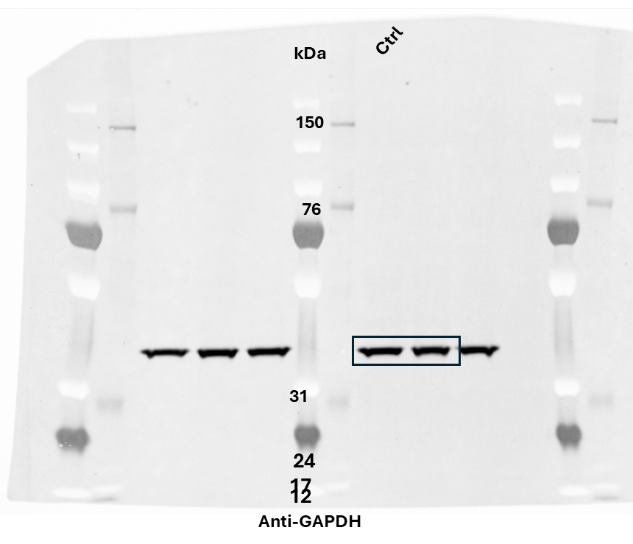
**


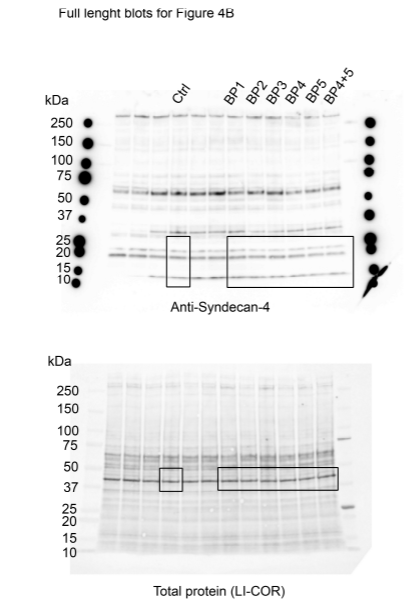


**
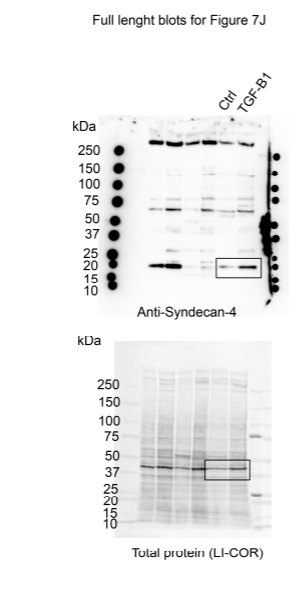
**

**Full length blot for Figure S3A**

**
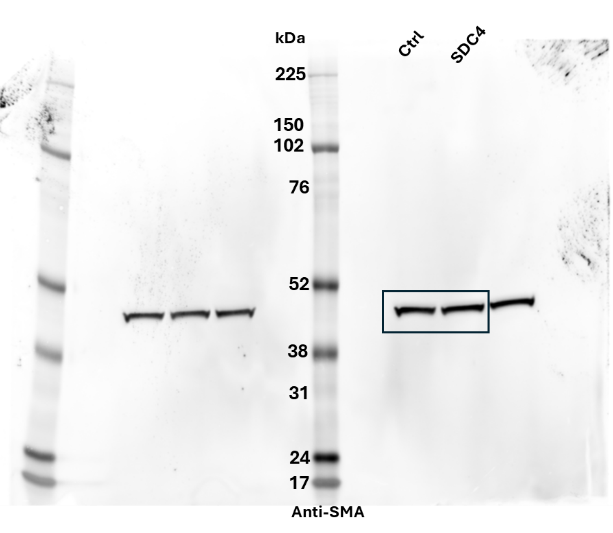

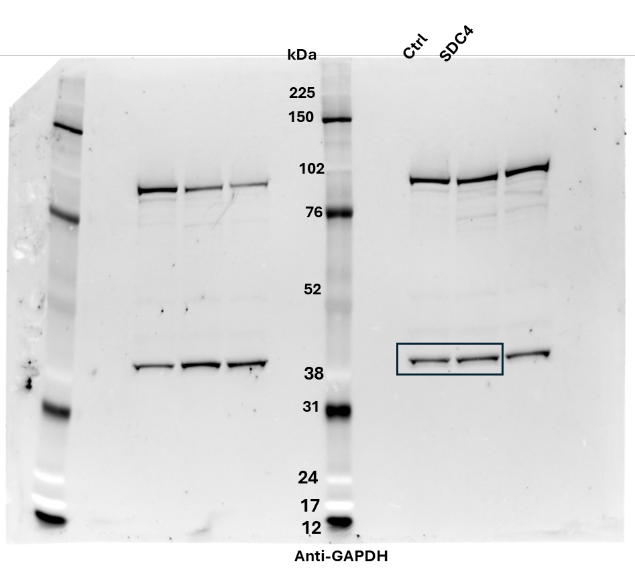
**

**Full length blot for Figure S4A**

**
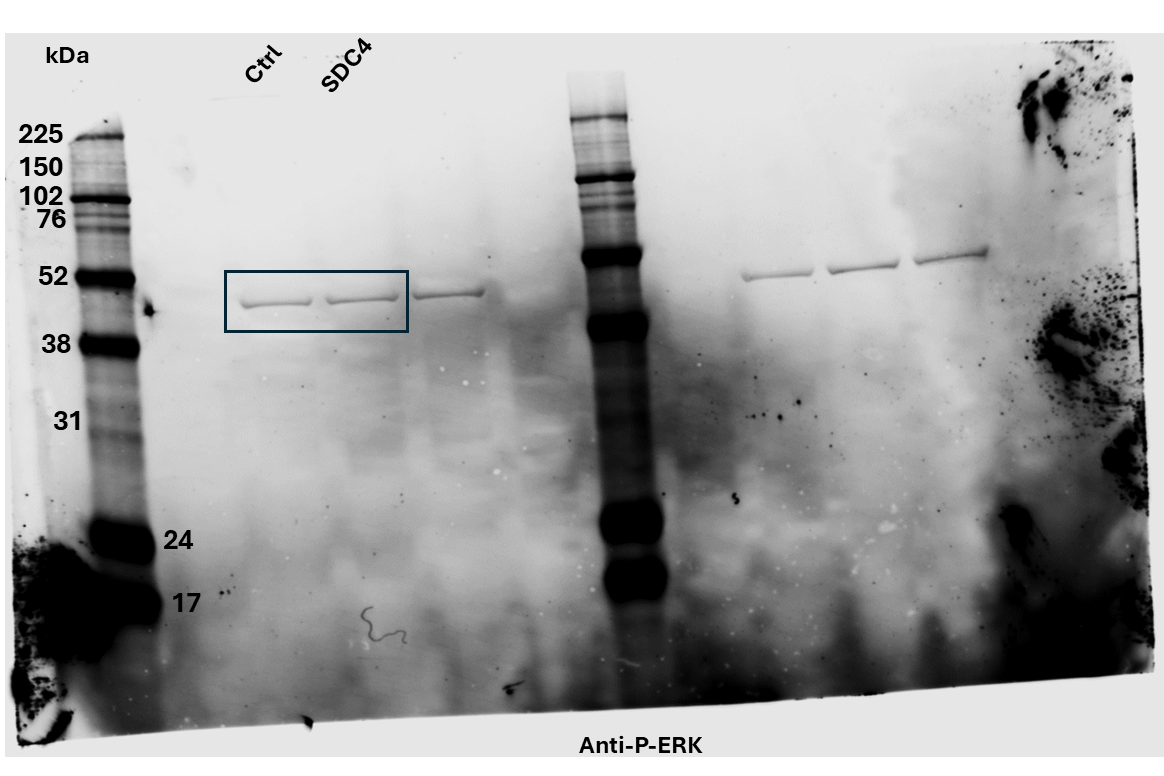

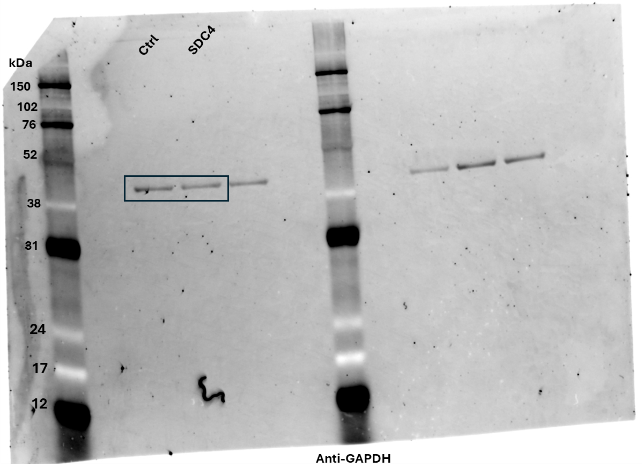
**

**
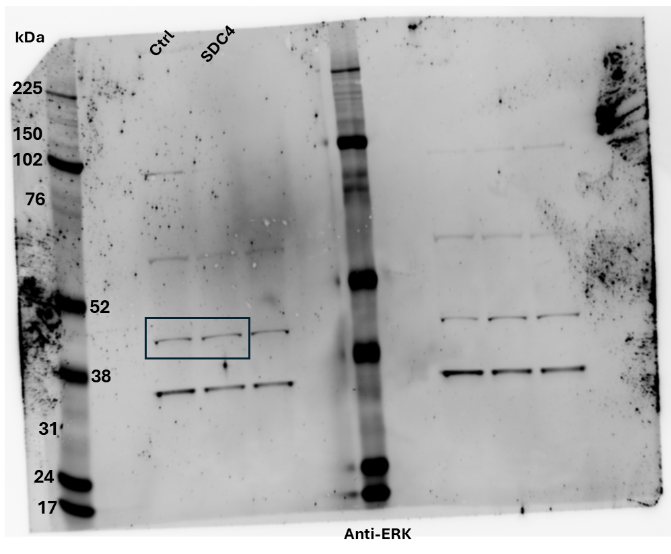

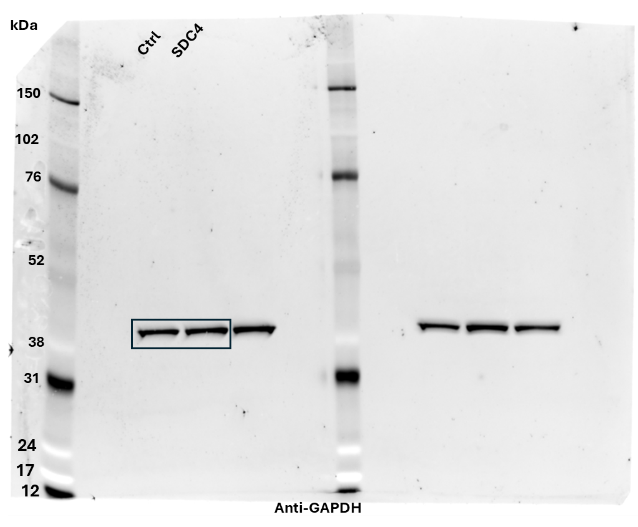
**

**Full length blot for Figure S4B**

**
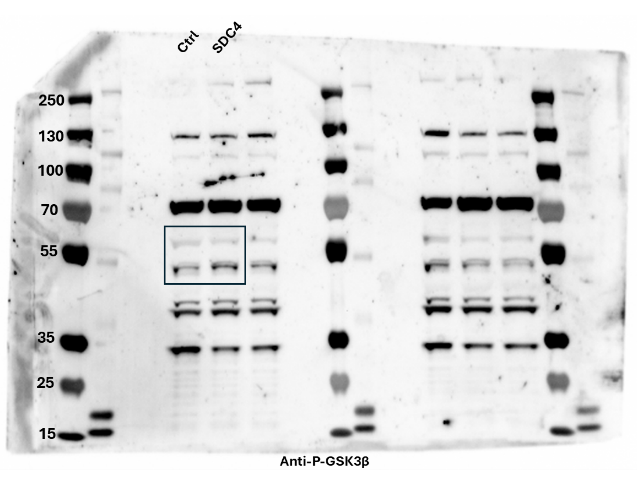

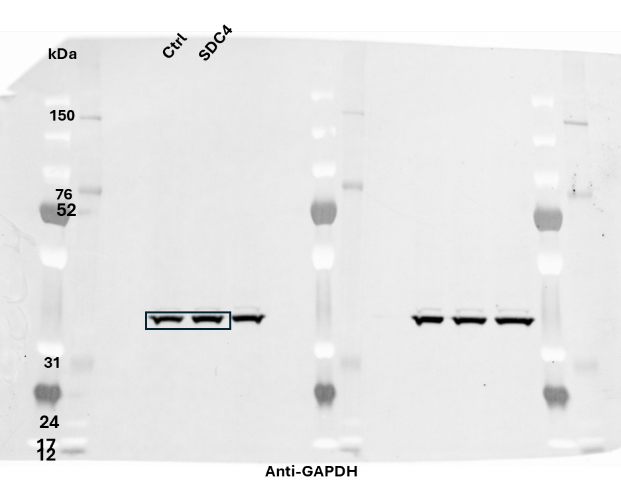
**

**
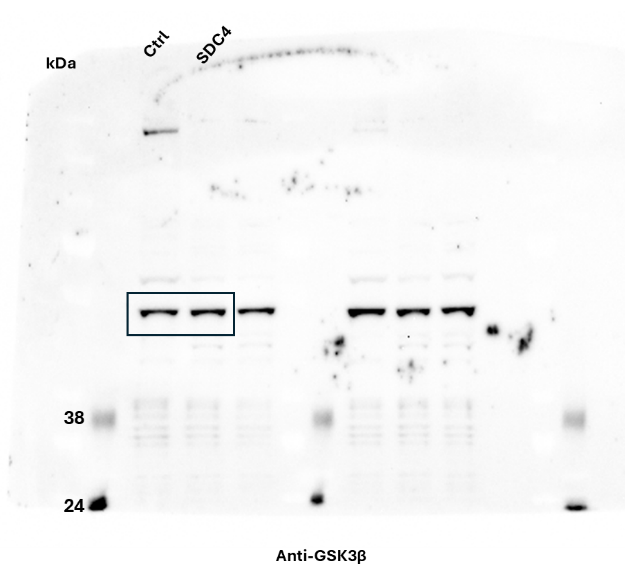

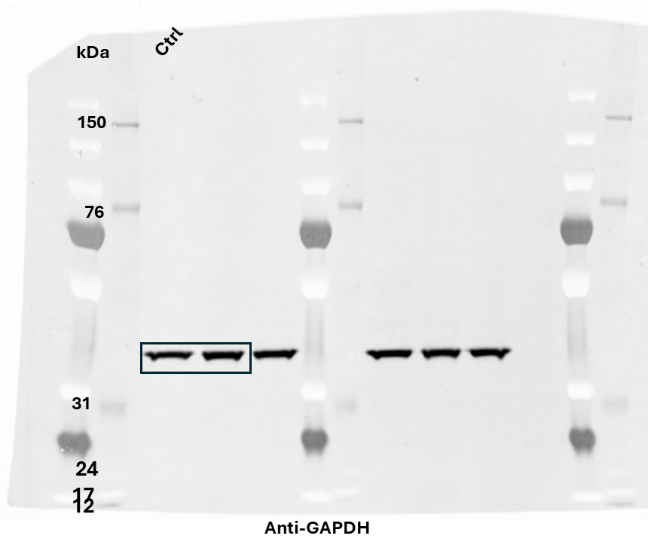
**
